# Understanding Experiences of and Unmet Needs in Online Searches for Menopause Information: An Exploratory Survey

**DOI:** 10.2196/75335

**Published:** 2025-10-01

**Authors:** Erin Lucy Funnell, Freya McConnell, Nayra A Martin-Key, Leyao Qian, Kathryn Babbitt, Sabine Bahn

**Affiliations:** 1 Cambridge Centre for Neuropsychiatric Research Department of Chemical Engineering and Biotechnology University of Cambridge Cambridge United Kingdom; 2 Department of Psychology University of Cambridge Cambridge United Kingdom

**Keywords:** menopause, perimenopause, online, internet, information

## Abstract

**Background:**

Menopause is a significant time in a woman’s life, but only recently has there been an open discussion about it in the media, workplaces, and general society. With increasing frequency, women are using the internet to research menopause, making it essential that online sources provide safe, high-quality, and relevant information.

**Objective:**

This study aimed to investigate the current state of the online information landscape for menopause from the perspective of information seekers, exploring (1) information-seeking behavior and (2) perceptions of online resources for menopause.

**Methods:**

A 10- to 15-minute online survey was conducted asking about the respondents’ use of and opinions about online resources specifically for menopause. We distributed the survey via social media, email, and word of mouth. Quantitative data were explored using means and frequencies. Group differences between menopausal groups were analyzed using chi-square, Fisher exact, or Kruskall-Wallis tests as appropriate. Qualitative data were analyzed using data-driven thematic analysis.

**Results:**

Data from 627 participants were analyzed (early perimenopause: n=171, 27.3%, late perimenopause: n=125, 19.9%, postmenopause: n=262, 41.8%, and surgical menopause: n=69, 11%). The majority of respondents had used the internet as a source of information (581/627, 92.7%), with the internet being the first choice of information source (489/581, 84.2%). The most searched-for information online was about menopause symptoms (479/581, 82.4%), menopause treatment options (442/581, 76.1%), and self-help tips or strategies (318/581, 54.7%). The majority of participants trusted online information to some extent (615/627, 98.1%), with many also considering online information accurate to some extent (555/627, 88.5%). Many participants reported finding some but not all of the information they were looking for online (379/581, 65.2%). Thematic analysis revealed 10 themes related to information quality and accessibility and sought-after information (eg, symptom specifics, treatment, and nonformal management strategies). Analysis also indicated that information is lacking for several groups, including those in medically induced or surgical menopause.

**Conclusions:**

The study showed that online informational resources are widely accessed and widely perceived as useful and trustworthy. However, it is crucial that the quality of online information is evaluated, especially considering the large number of users who rely on it as their first or only informational source. Online searches were usually performed to find information related to symptoms, treatment, and self-help recommendations, with differences in search behaviors observed across menopausal stages and groups, highlighting the need for tailored informational resources. Thematic analysis revealed gaps in the provision of online information both in terms of content and quality. Participants noted a lack of comprehensive symptom information, inadequate information for groups such as those experiencing medical or surgical menopause, and concerns about outdated content and a lack of source transparency. Future research with more diverse samples is needed to better understand variations in online health information-seeking behaviors across groups.

## Introduction

### Background

Menopause is defined as the time at which there has been no menstrual period for 12 months. Menopause may be naturally occurring or may be medically or surgically induced [1]. Natural menopause occurs due to the decline of ovarian follicular function, typically occurring between the ages of 45 and 55 years [2]. It is important to note that menopause can occur at any age, and the age at which a specific woman experiences menopause cannot be predicted.

Experiencing menopause before the age of 40 years is known as premature menopause and may be caused by several physical conditions, although in some women causal factors cannot be identified [2]. The perimenopause, or menopause transition, is the period before menopause where the frequency or duration of menstruation may change, and menopause symptoms may be present. On average, this occurs 4 years before menopause [3]. Menopause and perimenopause are associated with a wide range of both physical and mental health symptoms, the severity of which varies from person to person. Symptoms are wide-ranging, with some more well-known than others. Vasomotor symptoms, including hot flashes and night sweats, are common and are seen in as many as 80% of menopausal individuals [4]. The effects of menopause can cause huge difficulties in multiple domains of daily life, including professionally, personally, and with regard to health and well-being. As many as 31% of women have to take time off work due to their symptoms [5], with some having to give up work completely. In addition, 74% of women blame menopause for the breakdown of their marriage, and 67% of women report an increase in arguments and domestic abuse in relation to menopause [6].

It is clear that menopause is complex, potentially resulting in a variety of interpersonal problems, health difficulties, and poorer well-being. Menopause and the menopause transition require decisions to be made about when to reach out for help and which treatment intervention to use. Previous work has found that women feel unprepared for menopause and think that the early education of women and the wider society about menopause is lacking [7]. Research has also demonstrated that technology is perceived as a beneficial resource to support individuals facing menopause, being broadly used for 4 health-related purposes: connecting and sharing, information seeking, monitoring and reflection, and self-care [8]. Research also demonstrates the widespread use of the internet to gather information related to women’s health, with data from the United Kingdom revealing that 71% of women choose to use Google to find information about menopause, and 69% of women use other search engines or blogs as an additional source of information [9]. This is compared to only 59% of women who consult their general practitioner (GP) or other health care professionals (HCPs) to obtain this information. This highlights the need for safe, relevant, comprehensive, and clinically accurate information to be easily found online [9]. This widespread use of the internet as a source of information is also observed specifically for information about menopause, with a reported use rate of 74% in previous work [10].

To date, research has been focused on which informational sources women are using, online or otherwise, with limited research into the specifics of the information being sought. Some previous research identified the need for information regarding symptoms and treatment information [11,12], as well as the risks of secondary health conditions, such as cancer [12]. To our knowledge, there have been no studies addressing the specific informational needs of menopausal women in the United Kingdom when searching online. This is important, as the Menopause Priority Setting Partnership Steering Group has highlighted the need for research to identify ways to help people prepare for the perimenopause and menopause, as well as to develop awareness of when to seek help, with one potential avenue to this improved preparation and knowledge being online resources [13].

### Objectives

This study focuses on this knowledge gap, as without this information, the creation of useful online informational resources for menopausal women is not feasible. More specifically, the aims of this study were to (1) explore the information-seeking behavior of women in menopause and the menopause transition and (2) explore the perceptions of online informational resources for menopause. These insights will also be valuable in the design of content and delivery of future online resources. Relevancy of information is potentially as important as its quality, and what is classed as relevant may vary in women who are at different stages of menopause. Therefore, this study also considered the influence of specific menopausal stages on online searching behaviors, and perceptions of available online information resources.

## Methods

### Overview

The research team comprised a psychology and behavioral sciences undergraduate student (LQ), a master’s-level chemical engineering and biotechnology student (FM), 2 research assistants (ELF and KB), a senior research associate (NAM-K), and a practicing psychiatrist and professor of neurotechnology (SB). All authors involved in the qualitative analysis identify as female. All authors involved in the qualitative analysis received training in or have experience conducting thematic analysis.

The Checklist for Reporting Results of Internet E-Surveys for the current manuscript is provided in Multimedia Appendix 1.

### Materials

A novel 10- to 15-minute online open survey was delivered using Qualtrics XM (Qualtrics International Inc). The survey consisted of approximately 77 questions; the flow of questions was adaptive, dependent on previous answers. All but 3 free-text questions were closed questions and had prespecified answer options, including single choice, multiple choice, and Likert scale formats. A single question is presented per page of the survey. All questions had a back button, meaning that participants could rereview and amend their answers if necessary. All closed questions were marked as required preventing participants from proceeding to the next question without entering an answer. Free-text questions were optional.

Questions were developed by FM with input from ELF and NAM-K, with some questions, particularly those related to sources of information and information sought, adapted from other similar studies [7,12,13]. All questions were reviewed and approved by a practicing psychiatrist (SB). All aspects of the study, including the consent form, participant information sheet, and questionnaire, were completed in the same online survey. The survey comprised quantitative and qualitative questions on sociodemographic characteristics (eg, age, gender, and ethnic group), menopause symptom presence and severity, and online informational source usage (ie, participants’ experiences with and interest in using online informational sources for menopause). Questions about online informational source use focused on what type of information women search for online about menopause (eg, treatment options, health risks, or more general advice) and what type of source they feel most comfortable using. Motives behind using the internet to obtain information were explored, along with views on the currently available online resources. Usability testing (ie, testing all the adaptive logic, instruction clarity, response option functionality, and overall user experience) of the Qualtrics survey was performed by FM, ELF, and NAM-K. This process also provided an estimate of the time required to complete the survey, which was used to inform participants during the recruitment process. Responses to the consent form and survey were automatically saved by the Qualtrics software, and completeness checks (ie, percentage of the survey answered) were also completed by the survey software. For the current analysis, only questions related to sociodemographic characteristics and online informational source use were included. To see the survey questions, refer to Multimedia Appendix 2.

### Participants

Recruitment was carried out between January 5, 2024, and January 15, 2024, using digital convenience sampling. Participants were required to be aged ≥18 years, living in the United Kingdom, not have been pregnant or breastfeeding in the past 12 months, and confirm that they were in menopause or the menopause transition and experiencing at least 1 related symptom (eg, irregular or absent periods, hot flushes, and night sweats). Participants were required to self-define which stage of menopause or the menopause transition they were in. Definitions of different menopause stages provided in the survey were based on the Staging of Reproductive Aging Workshop guidelines [14,15]. Definitions of menopause stages provided in the survey were as follows: early perimenopause (significant change in the length or regularity of menstrual bleed not due to pregnancy, breastfeeding, stress, or a medical condition), late perimenopause (no menstrual bleeding in the past 3-11 months not due to pregnancy, breastfeeding, stress, or a medical condition), postmenopause (no menstrual bleeding in the past 12 months not due to pregnancy, breastfeeding, stress, or a medical condition), and surgical menopause (no bleeding in the past 12 months due to hysterectomy with 1 or 2 ovaries retained, or another medical intervention).

### Survey Distribution

The survey was disseminated using paid advertisements on social media, word of mouth, and emails sent to individuals who had previously participated in research carried out by the Cambridge Centre for Neuropsychiatric Research and had expressed interest in taking part in future studies. Recruitment materials specified that the study was focused on views of online resources for menopause. Paid advertisements via social media were delivered to specific populations based on self-reported gender and age to maximize the relevance of the recruitment materials to the target demographic.

### Statistical Analysis

Quantitative data were analyzed in Excel (version 2407; Microsoft Office 365) and SPSS (version 29.0.1.1; IBM Corp). Comparisons between binary categorical variables were analyzed with the chi-square or Fisher exact test for low-frequency data (<5 occurrences). Effect sizes are reported as Cramér V (φ_c_; small=0.10, medium=0.30, and large=0.50) [16]. For ordinal data with a Likert scale (ie, perceived accuracy and perceived trustworthiness), Kruskal-Wallis *H* tests were used, with post hoc Dunn tests subject to the Bonferroni correction for multiple comparisons conducted where appropriate.

The framework by Braun and Clarke [17] for thematic analysis was used for analysis of the open text data. A data-driven approach was adopted. Any free-text data that included “no” (or synonyms), “not applicable” (or synonyms), or responses that were considered ambiguous or nonspecific by both authors were labeled as not applicable and excluded from thematic analysis.

Before writing the codebooks, 1 author (ELF) read the open text data multiple times to gain familiarity with the data. During the codebook writing process, any responses that contained null data (eg, “no,” “none,” “N/A,” or synonyms) were labeled as “not applicable” by ELF. The codebook was reviewed by a second author (NAM-K), who gave feedback to maximize the comprehensiveness of the codebook. Open text data were coded against the codebook under blinded conditions by 2 authors (FM and LQ) and reviewed by a third author (ELF). While data were being coded, free-text responses were reviewed independently by 2 authors (FM and LQ) to determine their suitability for thematic analysis. Responses were labelled as “not applicable” and excluded if they met any of the following predefined criteria: (1) were off-topic or unrelated to the survey prompt or (2) were ambiguous, overly vague, or lacked sufficient detail for meaningful interpretation without avoiding the potential for assumptions to be made by the research team regarding the meaning (eg, “can’t say” and “unsure”). Responses were removed only when both authors agreed that they met the exclusion criteria. As part of criteria for exclusion number 1, positive responses such as “I found enough...” (n=9) were excluded because, given the negatively framed prompt, similar positive experiences may have gone unreported and including only a few such responses could introduce inconsistency. The coding allocations were unblinded and compared, with any discrepancies being discussed until an agreement was reached. After the coding allocation was complete and finalized, the codes were organized into themes by 2 authors (FM and LQ) under blinded conditions. Each author reviewed the codes and grouped them into initial themes based on patterns and conceptual similarity. The theme lists generated were then compared, with any discrepancies being discussed (FM, LQ, ELF, and KB) until a final theme list was agreed upon. This process involved iterative discussions to refine the scope and definitions of each theme, ensuring that themes were clearly defined, distinct, and grounded in the data. Please note that the illustrative quotes provided as examples of any codes identified have not been revised in any way. Therefore, any spelling or grammatical errors are as provided by the participants.

### Ethical Considerations

Ethics approval was awarded by the Cambridge Psychological Research Ethics Committee (PRE.2023.124). Participants were required to give informed consent to participate electronically before the main body of the survey began. Participation was completely voluntary. Data collected were anonymized using randomly generated participant IDs by the survey software and stored on password-protected devices. In exchange for participation, participants were invited to enter a draw for the chance to win one of three £50 (US $64.72) gift cards.

## Results

### Survey Uptake

A total of 1843 responses were recorded for the survey. In total, 774 participants consented to take part in the study, with 628 complete responses (ie, 100% completion rate). One response was omitted from further analysis because the respondent disclosed that they had given false demographic information, leaving a final sample of 627 participants (view rate: 627/1843, 34.02%, completion rate: 627/774, 81%, early perimenopause: 171/627, 27.3%, late perimenopause: 125/627, 19.9%, postmenopause: 262/627, 41.8%, and surgical menopause: 69/627, 11%).

### Demographics

Full sociodemographic characteristics of the overall sample are reported in Table 1 (sociodemographic characteristics for each of the menopause stages can be found in Table S1 in Multimedia Appendix 3). The average age of participants was 52.40 (SD 4.67; range 31-71) years. The majority of the sample were White (594/627, 94.7%), identified as female (617/627, 98.4%), were in paid employment (446/627, 71.1%), and were married or in a civil partnership (379/627, 60.5%). Moreover, 48.8% (n=306) held at least an undergraduate degree, 42.4% (n=266) had an annual household income before tax of at least £45,001 (US $60,232.50), and 40.34% (n=253) of the participants reported having preexisting conditions with symptoms that may overlap with the symptoms of menopause.

**Table 1:** Sociodemographic characteristics (N=627)^a^.

Sociodemographics		Respondents, n (%)
**Gender**		
	Woman	617 (98.4)
	Man	2 (0.3)
	Nonbinary	1 (0.2)
	Other	5 (0.8)
	Prefer not to answer	2 (0.3)
**Ethnicity**		
	Asian or Asian British	14 (2.2)
	Black, Black British, Caribbean, or African	16 (2.6)
	Mixed or multiple ethnic groups	3 (0.5)
	White	592 (94.7)
**Marital status**		
	Single	86 (13.7)
	Married or civil partnership	379 (60.4)
	Cohabiting	74 (11.8)
	Separated	23 (3.7)
	Divorced	41 (6.5)
	Other	18 (2.9)
	Prefer not to answer	6 (1)
**Education**		
	Below GCSE^b^ or equivalent	37 (5.9)
	GCSEs, Scottish Higher, or equivalent	106 (16.9)
	A-Levels^c^, International Baccalaureate, or equivalent	133 (21.2)
	Undergraduate degree	152 (24.2)
	Postgraduate degree	154 (24.6)
	Other	26 (4.2)
	Prefer not to answer	19 (3)
**Employment^d^**		
	Employed full-time	289 (46.1)
	Employed part-time	149 (23.8)
	Self-employed	71 (11.3)
	Parental leave	26 (4.2)
	Student	4 (0.6)
	Retired	39 (6.2)
	Volunteer	14 (2.2)
	Unemployed	44 (7)
	Prefer not to answer	11 (1.8)
**Annual household income (before tax;** **£ [US $])**		
	<15,000 (US $20,226)	49 (7.8)
	15,001-25,000 (US $20,227.35-$33,710.00)	69 (11)
	25,001-35,000 (US $33,711.35-$47,194.00)	73 (11.6)
	35,001-45,000 (US $47,195.35-$60,678.00)	73 (11.6)
	45,001-55,000 (US $60,679.35-$74,162.00)	69 (11)
	55,001-65,000 (US $74,163.35-$87,646.00)	49 (7.8)
	65,001-75,000 (US $87,647.35-$101,130.00)	37 (5.9)
	75,0001-85,000 (US $101,131.35-$114,614.00)	33 (5.3)
	>85,001 (US $114,615.35)	78 (12.4)
	Prefer not to answer	97 (15.5)

^a^Total percentage may exceed 100%, as participants were able to select >1 type of employment.

^b^GCSE: General Certificate of Secondary Education.

^c^A-Level: Advanced Level.

^d^Total percentage may exceed 100%, as participants were able to select >1 type of employment.

### Use of the Internet

**Figure 1:**
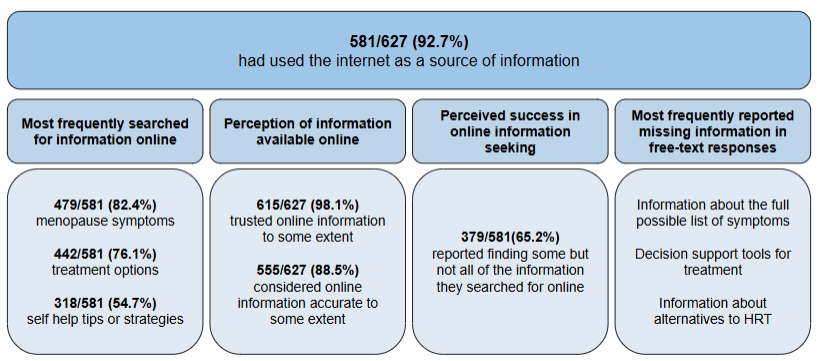
Summary of data related to the most frequently searched for information, the perception of information available online, the perceived success in online information seeking, and missing information. Missing information is drawn from the qualitative data (N=394) and reflects the most frequently identified codes in the dataset. HRT: hormone replacement therapy.

The vast majority of respondents had used the internet as a source of information (overall: 581/627, 92.7%, early perimenopause: 162/171, 94.7%, late perimenopause: 112/125, 89.6%, postmenopause: 242/262, 92.4%, and surgical menopause: 65/69, 94%). Of those who did not use the internet as a source of information (overall: 46/627, 7.3%), the most common reason for not using the internet provided was not knowing where to start looking (17/46, 40%; Table S2 in Multimedia Appendix 3). Figure 1 provides a summary of data related to the focus of searches for information, the perception of online information, satisfaction with online information, and perceived missing information.

Of those who endorsed looking for information on the internet, the majority used the internet as their initial source of information (overall: 489/581, 84.2%, early perimenopause: 144/162, 88.9%, late perimenopause: 88/112, 78.6%, postmenopause: 204/242, 84.3%, and surgical menopause: 53/65, 82%). Table 2 provides a summary of informational resources accessed online. No group differences were observed regarding the type of information resources accessed. Informational resources on the internet were most frequently found via a Google Search (511/581, 88%).

**Table 2:** Information sources accessed online. Total percentages may exceed 100% because multiple information sources could be selected. Percentages were calculated by dividing the frequency of each code by the total number of responses (overall: n=581, early perimenopause: n=162, late perimenopause: n=112, natural menopause: n=242, and surgical menopause: n=65).

Information source	Overall, n (%)	Early perimenopause, n (%)	Late perimenopause, n (%)	Natural menopause, n (%)	Surgical and medical menopause, n (%)
NHS^a^ online and NHS inform	447 (76.9)	130 (80.3)	82 (73.2)	180 (74.4)	55 (84.6)
Websites reporting other people’s experiences	186 (32)	56 (34.6)	38 (33.9)	76 (31.4)	16 (24.6)
Websites suggesting in-person support options	103 (17.7)	27 (16.7)	35 (22.3)	40 (16.5)	11 (16.9)
Charities	262 (45.1)	80 (49.4)	53 (47.3)	96 (39.7)	33 (50.8)
Web sites aimed at HCPs^b^	143 (24.6)	43 (26.5)	24 (21.4)	56 (23.1)	20 (30.8)
Social media	359 (61.8)	98 (60.5)	80 (71.4)	145 (59.9)	36 (55.4)
Scientific literature	73 (12.6)	18 (11.1)	9 (8)	33 (13.6)	13 (20)
Online news sites with reports about menopause	205 (35.3)	55 (34)	37 (33)	86 (35.5)	27 (41.5)
Other	44 (7.6)	13 (8)	10 (8.9)	17 (7)	4 (6.2)

^a^NHS: National Health Service.

^b^HCP: health care professional*.*

### Other Information Sources

Most online information seekers also sought information elsewhere (overall: 439/581, 75.6%), with a similar proportion between menopause stages (early perimenopause: 126/162, 77.8%, late perimenopause: 88/112, 78.6%, postmenopause: 174/242, 71.9%, and surgical menopause: 51/65, 78%).

Most of these had used a secondary source after using the internet as the primary source of menopause information (overall: 353/581, 60.8%, early perimenopause: 110/162, 67.9%, late perimenopause: 65/112, 58%, postmenopause: 139/242, 57.4%, and surgical menopause: 39/65, 60%; Table S3 in Multimedia Appendix 3). The most frequently cited reason for using a second source of information after the internet was to obtain as much information as possible (183/353, 51.8%), followed by wanting verification that the information found online was correct (164/353, 46.5%; Table S4 in Multimedia Appendix 3). A higher proportion of the surgical menopause group reported using a secondary source of information, as they could not find the information they were looking for online (9/39, 23%) compared to the early perimenopause group (8/110, 7.3%; χ^2^_3_=8; *P*=.045; φ_c_=0.15). A larger proportion of the late perimenopause group (40/65, 62%) wanted verification that the information found online was correct and trustworthy, compared to the postmenopause group (55/139, 39.6%; χ^2^_3_=9.8; *P*=.02; φ_c_=0.17). A larger proportion of the early perimenopause group considered the information available online overwhelming (45/110, 40.9%) than the postmenopause group (34/139, 24.5%; χ^2^_3_=8.4; *P*=.04; φ_c_=0.15).

Of those who had not used a secondary informational source in addition to the internet, the most frequent reason for not doing so was not thinking symptoms were severe enough for further help-seeking (57/136, 41.9%) and that they found enough information online (55/136, 40.4%; Table S5 in Multimedia Appendix 3). The surgical menopause group had a higher proportion of individuals citing not using additional noninternet sources due to not trusting an HCP (3/14, 21%) compared to the postmenopause group (1/65, 2%; χ^2^_3_=11.9; *P*=.008; φ_c_=0.27).

The source of information most frequently used other than the internet was a GP (353/439, 80.4%; Table S6 in Multimedia Appendix 3). A higher proportion of the surgical menopause group had contacted a gynecologist compared to any other menopause stage (Table 2; χ^2^_3_=28.6; *P*<.001; φ_c_=0.26). Use of a support group was more common in women in the surgical menopause group (χ^2^_3_=8.6; *P*=.04; φ_c_=0.14). The early perimenopause group endorsed consulting a pharmacist less than any other group (Table 2; χ^2^_3_=10.1; *P*=.02; φ_c_=0.15).

### Common Online Search Topics

Table 3 shows what information participants most frequently endorsed looking for online (Table S7 in Multimedia Appendix 3). Information about menopause symptoms (479/581, 82.4%), menopause treatment options (442/581, 76.1%), and self-help tips or strategies (318/581, 54.7%) were endorsed as the most frequent online searches. Of the 33 participants who endorsed searching for information about surgical menopause, 28 (86%) were in surgical menopause. Searches related to pregnancy were more frequent in the late perimenopause group (6/112, 5.4%) than in the postmenopause group (3/242, 1.2%; χ^2^_3_=8.2; *P*=.01; φ_c_=0.12). In addition, a larger proportion of the late perimenopause group (21/112, 18.8%) had searched online for information about private care than the postmenopause group (21/242, 8.7%; χ^2^_3_=8.3; *P*=.04; φ_c_=0.120). A larger proportion of the surgical menopause group had searched online for information about surgical menopause (28/65, 43%) compared to all other groups (early perimenopause: 2/162, 1.2%, late perimenopause: 1/112, 0.9%, and postmenopause: 2/242, 0.8%; χ^2^_3_=191.1; *P*<.001; φ_c_=0.57). A higher proportion of participants in the late perimenopause group (37/112, 33%) and postmenopause group had searched online for general advice for menopause (ie, “how to...;” 70/242, 28.9%) compared to the surgical menopause group (9/65, 14%; χ^2^_3_=8.9; *P*=.03; φ_c_=0.12).

**Table 3:** Frequency of topics around menopause searched for (N=581).

Topic searched for	Overall, n (%)
Causes of menopause	69 (11.9)
Menopause treatment side effects	312 (53.7)
Health risks related to menopause	275 (47.3)
Pregnancy and menopause	16 (2.8)
Menopause consequences (eg, “difficulties with...”)	283 (48.7)
Menopause symptoms	479 (82.4)
Menopause treatment options	442 (76.1)
Private care options	65 (11.2)
Support groups	117 (20.1)
Surgical menopause	33 (5.7)
General advice (ie, “how to...”)	155 (26.7)
Self-help tips or strategies	318 (54.7)
Other	32 (5.5)

Regarding online searching for information about health-related risks related to menopause (Table S8 in Multimedia Appendix 3), the most searched for possible health risk was osteoporosis (188/275, 68.4%). A higher proportion of participants in the surgical menopause group had searched online for information about osteoporosis (32/36, 89%) than participants in the early (52/80, 65%) and the late perimenopause group (31/56, 55%; χ^2^_3_=12.1; *P*=.007; φ_c_=0.21). A higher proportion of participants in the late perimenopause group (26/56, 46%) searched online for information about urinary tract infections than the early perimenopause (17/80, 21%) and postmenopause groups (23/103, 22.3%; χ^2^_3_=13.8; *P*=.003; φ_c_=0.22).

The treatment information most frequently searched online (Table S9 in Multimedia Appendix 3) was about transdermal hormone replacement therapy (HRT; 289/442, 65.4%), followed by oral HRT (183/442, 41.4%), vaginal HRT (118/442, 26.7%), lifestyle changes (171/442, 38.7%), and testosterone (108/442, 24.4%). A higher proportion of individuals in the surgical menopause group had searched online for information about lubricants (15/47, 32%) than all other groups (early perimenopause: 13/132, 9.8%, late perimenopause: 7/89, 8%, postmenopause: 18/174, 10.3%; χ^2^_3_=20.1; *P*<.001; φ_c_=0.213). A higher proportion of individuals in the late perimenopause group had searched online for information about complementary therapies (30/89, 34%) than the early perimenopause (18/132, 13.6%) and postmenopause (34/174, 19.5%) groups (χ^2^_3_=13.2; *P*=.004; φ_c_=0.173).

The treatment side effects most frequently searched for online (Table S10 in Multimedia Appendix 3) were weight gain (187/312, 59.9%), mood changes (178/312, 57.1%), and risk of cancer (173/312, 55.5%). Group differences were observed for online searches about irregular bleeding (χ^2^_3_=17.8; *P*<.001; φ_c_=0.24), with a higher proportion of participants in early (31/93, 33%) and late (21/58, 36%) perimenopause endorsing searching for online information about irregular bleeding than participants in postmenopause (22/130, 16.9%) or surgical menopause (2/31, 6%) groups. Participants in the early (26/93, 28%) and late (19/58, 33%) perimenopause groups were proportionally more likely to look for information about dizziness as a treatment side effect than those in the postmenopause (18/130, 13.9%) and surgical menopause (9/31, 29%; χ^2^_3_=13.9; *P*=.003; φ_c_=0.21) groups.

In terms of general advice searched for online (Table S11 in Multimedia Appendix 3), the majority wanted information about how to ask for support generally (66/155, 42.6%) or information about how to talk to an HCP about menopause (65/155, 41.9%). No group differences were observed for general advice searching online.

### Perceptions of the Quality of the Internet as a Source of Information

Most participants perceived information available in online sources as accurate to some extent (555/627, 88.5%), with the largest proportion considering the sources available online to be mostly accurate (304/627, 48.5%; Table S12 in Multimedia Appendix 3). There was a significant difference in the perceived accuracy of online information across menopause stages (H_3_=8.827; *P*=.032). Post hoc tests revealed a significant difference in perceived accuracy between the postmenopause and surgical menopause groups (*P*=.043), with those in the postmenopause group considering the information available online to be of a higher accuracy.

Most participants trusted online sources of information to some extent (615/627, 98.1%), with the largest proportion reporting moderate trust (390/627, 62.2%; Table S12 in Multimedia Appendix 3)*.* The online informational resources considered the most trustworthy were the National Health Service (NHS) or NHS Inform websites (490/627, 78.2%) and the websites of charities (401/627, 64%; Table S12 in Multimedia Appendix 3). No significant differences in trustworthiness were observed between menopause stages.

Most participants who had looked online for information related to menopause found some but not all the information they were looking for online (379/581, 65.2%), with only 4.5% (26/581) reporting that they had not found what they were looking for online (Table S3 in Multimedia Appendix 3). No differences were observed in perceived comprehensiveness between the menopause stages.

### Qualitative Analysis

In total, 593 qualitative responses were provided. Of these, 199 (33.6%) were labeled as “not applicable” and removed. A final dataset of 394 (66.4%) responses was analyzed. Thematic analysis revealed 68 codes organized into 10 themes (Table S13 in Multimedia Appendix 3 provides the complete list of identified codes with their code frequencies).

### Information Quality and Accessibility

Generally, perceptions of the quality of online resources expressed in free-text responses were negative. Participants who commented on information quality indicated that they perceived available information to be generic. In addition, there were concerns about the accuracy of the information available online, with it being perceived as outdated and lacking transparency regarding the source of the information presented. Some participants commented on their perception that some online content frame as menopause information was instead deceptive advertising (“I feel like I get sucked into social media ads targeted at 50+ year old women trying to sell me dubious supplements.”). For some participants, this resulted in a sense of distrust of the information available online.

Regarding accessibility, participants commented on a perceived lack of research focused on menopause. There was also a request for improved dissemination of research online (“I’d like to be more familiar with research into the menopause.”)*.*

In addition, there were reports that information is inaccessible because it is not written in an easy-to-understand way (“I would like it all explained in a simpler way, as having brain fog made it hard for me to take it in.”) or that websites are difficult to use. Similarly, some participants expressed that the information available online was overwhelming. As a potential solution, participants called for “One site that has all the information, instead of being sent from site to site,” such as a centralized menopause information hub.

### Treatment

Thematic analysis revealed a request for easier-to-understand information and guidance for treatment pathways and menopause interventions. Some participants specifically asked for decision-support tools, such as flowcharts (“Types of HRT—a nice flow-chart to help me choose”). This included a request for information about the safety of menopause management options, including long-term outcomes (“I have very little clue about what my options are together with what the side effects may be for me”). In addition, participants mentioned needing more information about the association between menopause treatments, such as HRT, and preexisting or comorbid conditions, such as cancer (“Specific info & treatments for someone who has had triple negative breast cancer”). Participants also noted the need for diversification of existing treatment guidelines to provide tailored information on menopause management for specific subgroups, such as those experiencing medically or surgically induced menopause (“There is no NICE guidance on risks of HRT post surgery for endometrial cancer and very little research”).

Specifically regarding HRT, participants noted that there is misinformation related to HRT online (“Accurate information about the health benefits of [HRT] and removal of the hugely out of date and debunked ‘scare stories’ about HRT.”), revealing a request for clearer and more balanced information. Free-text responses also requested more information related to specific types of HRT, such as testosterone (“More evidence of adding testosterone to the HRT options and it's benefits”), as well as alternatives to HRT.

Free-text responses also requested information regarding alternatives to HRT for those who cannot or do not wish to use HRT (“Treatment other than medication for people who can't use HRT”). Relatedly, other participants sought information about the consequences of not taking any menopause treatment (ie, letting menopause happen “naturally”; “What if you don't do anything at all and let it all happen naturally, what are the consequences of this”).

### Groups Lacking Information

Free-text data revealed a perception of information missing for certain groups, including HCPs (“Resources to take to your GP if they are not up to date on the menopause.”), workplaces (“More online resources for employers to access.”), individuals in medical or surgical menopause (“More recognition about surgical menopause and the impact of symptoms”), partners (“Menopause should be taught to both women and men. I had to make my husband read others experiences so that he understand my plight and the help I needed.”), and men and boys (“Educate boys and men, it's important”), with requests for informational resources tailored to these groups to maximize their relevance.

Conversely, some participants stated they held a perception that the information online is tailored for specific groups (eg, women who may be considered “healthy” or not have any existing or comorbid conditions), which can make the information feel inaccessible or irrelevant (“Much seemed geared towards ‘normal’ healthy women—ie, not fat like me, or with pre-existing depression.”).

### Symptom Specifics

Participants also requested more information about symptoms of menopause. This included the complete list of menopausal symptoms, including “atypical” symptoms, such as dental symptoms, dry eyes, and migraines (“finding concise information about the wide-ranging nature of symptoms is difficult”, “Medical professionals/NHS websites providing full lists of symptoms as some quite common ones aren’t included, e.g. itchy ears and bleeding gums.”). Relatedly, free-text data included requests for more comprehensive information on some domains of menopause symptoms, such as urogenital and sexual symptoms (“More information about sex, painful sex”) and mental health symptoms (“More information about the psychological symptoms and how to manage them. Most information is heavily swayed towards the physical symptoms. Not enough help or information about the psychological side.”).

Beyond the need for a complete list of symptoms, requests were made about information to help understand the chronicity *(*“There’s no information about what symptoms are permanent and what are transient.”) and prevalence of these symptoms (“Perhaps more data rather than the ‘you might experience’ just to get some idea about how prevalent particular symptoms are.”).

Participants also requested information on how to recognize or “spot” that you may be in perimenopause or menopause (“How to tell you are peri menopausal”), indicating a possible lack of clarity of how symptoms map onto the different menopause stages.

### Health Care Access and Support (Including Offline)

Participants requested more online resources to provide recommendations of when it may be appropriate to seek formal support from an HCP (“When to see GP”). In the free-text data, participants also expressed the importance of having access to menopause specialists (“To be able to speak to a gynaecologist rather than a GP”) and seeking information about how to gain a referral to such specialists (“How to refer oneself to specialist services”). In addition, participants requested recommendations on how to speak to an HCP about menopause, including how to self-advocate (“How to advocate for yourself when contacting a GP about menopause treatment”), noting the importance of accessing HCPs alongside online resources (“there should be medical professionals to provide more information, reassurance”).

### Other Support Outside of Formal Care

In terms of online information in relation to support outside of HCPs, participants in this study requested additional resources to guide them in gaining support in the workplace (“What are our rights re work support. How to get help when work becomes difficult [due] to symptoms”). Furthermore, support groups were mentioned as a useful support resource, with participants either expressing their benefits or requesting improved access to directories to find relevant support groups (*“*what I found most helpful was some facebook groups where women shared their experiences and stories as then there was always something you could relate to and understand how they’d managed or what advice they gave or had found etc.*”*).

### Lifestyle Support and Self-Management

Thematic analysis revealed an interest in online information regarding nonformal interventions to manage menopausal symptoms, including natural remedies, such as supplements and lifestyle changes (“naturally treat/support hormonal changes with the appropriate supplements”). In addition, some participants mentioned they wanted to see more online resources with information about food, diet, and exercise to manage symptoms of menopause or to lose weight (“Also more on exercising through menopause”). Free-text responses also included requests for reviews of supplements and other products specifically designed for use in menopause (“Reviews of natural supplements etc”).

### Menopause and Co-Occurring Conditions

Thematic analysis revealed requests for improved online information and guidance about the association between menopause and co-occurring conditions, including but not limited to chronic health conditions (“Specifications about how other chronic conditions can overlap with menopause”), psychological conditions (More advice about how the menopause can worse existing general anxiety disorder” and “[premenstrual dysphoric disorder] and it getting worse before menopause), and gynecologic conditions (“It is impossible to find any information for women who suffer with [polycystic ovary syndrome or PCOS] and how to manage this and menopause at the same time and they interact with each other. We are in desperate need of guidance and support for PCOS & Menopause”).

### Patient Empowerment

Thematic analysis also highlighted how online resources may be amended to empower patients. This included providing or enabling the sharing of narrative accounts in online spaces (“Personal stories of women coping and how they cope”) to understand the experiences and successful management strategies of other individuals in a similar circumstance. In a similar vein, some participants requested that online resources provide reassurance, conveying the benefits for comfort and encouragement (“I think reassurance that you will get through it as it’s quite daunting when you first go through it”).

### Perimenopause

Participants reported a perceived lack of online resources about perimenopause (“There isn’t much about early premenopause either. it would be helpful if NHS or other credible UK healthcare providers shared information on this. How long should one wait before taking any measures? It is ok to start later? What are the window of opportunity for taking which actions?”), making it difficult to obtain information about this stage of the menopause transition.

## Discussion

### Principal Findings

The aims of this study were to explore the online information-seeking behavior of women in menopause and the menopause transition. In addition, the study sought to gain insights into perceptions of online resources for menopause information. Most participants in this study had used the internet as a source of menopause information. The internet seems to be not only highly accessible but also acceptable, with it being frequently used as the first point of information gathering. It also seems that the internet is considered a high-quality resource for menopause information, with information perceived as mostly accurate and trustworthy. This is concerning, as reviews of online information for menopause have revealed varying quality [18]; however, given the pace with which websites can be updated, a more recent audit of quality and content is required. Reassuringly, the NHS website was the most frequently accessed resource for menopause information and thus is likely to be evidence based. However, qualitative analysis revealed a perception from the target audience for this resource that it may be considered out-of-date. Social media was the second most commonly accessed online resource for menopause information, revealing a considerable risk of misinformation. However, social media was not a resource that was frequently considered trustworthy. Therefore, this study highlights a potential need for guidance for those searching for menopause information online about how to differentiate high-quality, evidence-based content from potentially unreliable sources. Many participants had also sought information from other sources, with an NHS GP being the most frequently reported contact. Crucially, around 24.4% (142/581) of participants had only used the internet for information, not consulting any other information source. This further demonstrates the importance of validating the quality of information available online to tackle potential misinformation as well as maximizing the accuracy and utility of information found.

Information about menopause symptoms, menopause treatment options, and self-help tips or strategies was reported as most frequently looked for online. This reflects previous research investigating informational needs for menopausal individuals [10,11]. Other frequently searched topics were related to health, with participants endorsing having searched for information about menopause-related health risks as well as the interaction between menopause and other preexisting conditions. Interestingly, although information about menopause symptoms was the most commonly searched for information online, it seems that this information is also perceived to be not readily available. Many participants who provided a free-text response requested an improved provision of information online related to menopause symptoms, with there being a request for resources to provide a more extensive list of possible symptoms as well as information related to potential chronicity and duration of symptoms. Given the wide range of potential menopause symptoms and the distress that can accompany their onset [19], access to comprehensive information is vital. This is especially important in reducing the potential uncertainty or fear associated with the onset of unfamiliar or infrequently discussed menopause symptoms.

This study revealed that online search behavior varies considerably between menopausal stages. While the vast majority of participants, irrespective of menopause stage, made use of the internet as an informational resource, there were variations in the specific information that was searched for. For instance, those in the perimenopause searched online for information about general “how to” information and private care options, as well as complementary therapies. This may indicate that participants in this stage of menopause may have had poor experiences of formal care offered through the public sector and so were potentially motivated to seek information about alternative avenues for help and symptom management. This indicates the complexity that governs online searches for information about menopause, including but not limited to specific stages. Given this, future resources should aim to address the concerns and needs of different menopausal groups. This will involve in-depth co-design of materials, ensuring that a range of experiences and perspectives are captured and reflected in the resources generated. Beyond the menopausal stage, thematic analysis revealed a perception of a lack of information for other specific groups, such as those in medically induced or surgical menopause. Considering that surgical menopause is associated with poorer health-related quality of life outcomes [20] and more severe symptoms [21] compared to other menopausal groups it is crucial they are well-informed, but it appears that online information about menopause may not adequately address the unique needs of individuals who have undergone medically induced or surgical menopause. Therefore, there is a need to provide information that is relevant to the specific experiences of this subgroup.

Information about HRT was the most searched-for treatment information in this study, yet it is unclear whether these searches were a result of being offered HRT by an HCP, or if it was self-directed research. HRT is one of the most frequently prescribed treatments for the management of menopause symptoms, and therefore, these searches may indicate the goal of obtaining as much information as possible about this treatment option. However, there is evidence that some individuals have to self-advocate to access HRT and may be collecting relevant information to take to their HCP in this pursuit. Other than HRT, nearly a quarter of participants were looking for information about the use of testosterone for the management of menopause symptoms, with many free-text responses indicating an interest in this area. Testosterone is currently not licensed for use for menopause in the United Kingdom [22], with access to testosterone usually reserved for individuals presenting with reduced sex drive and when other HRT has not helped [23]. Although testosterone may not be widely prescribed, this study shows that there is obvious interest in its use. In fact, there was a 10-fold increase in the number of prescriptions of testosterone for women aged >50 years between 2015 and 2022 [24]. This increase may be associated with what has been described as the “Davina McCall effect,” with a sharp increase in the number of people receiving testosterone at the release of her first documentary about menopause [24]. A further increase was also observed after she released a second documentary that included information about testosterone treatment [24]. Apart from HRT, approximately 15% (68/442) of participants reported looking for information about antidepressants. According to the National Institute for Health and Care Excellence guidelines [23], if there is no independent diagnosis of major depressive disorder, prescription of antidepressants to treat the symptoms of low mood and anxiety related to menopause is not recommended. In practice, it has previously been reported that women experiencing low mood in menopause are prescribed selective serotonin reuptake inhibitors [25]. Prescriptions of antidepressants are seemingly perceived as inappropriate for menopause by those who have received them [26], and therefore, this search behavior may reflect uncertainty regarding the suitability and efficacy of this treatment option for menopause. The differing types of treatment information sought is also reflected in free text responses, with participants demonstrating interest in information regarding alternative interventions to HRT. Overall, the identified online search behaviors for menopause treatment information highlight a significant gap in accessible, comprehensive resources available on and offline. Therefore, there is a need for detailed and reliable information on the wide range of therapeutic interventions available, which could both support the navigation of the complex landscape of menopause treatment and empower informed decision-making, both before and after contact with an HCP. In fact, this was something frequently requested by participants, with calls for improved information online and interest in decision-support tools, such as flowcharts, which may assist individuals in identifying the most appropriate intervention for them. Ideally, these resources would also include details of interventions or self-help strategies that can be used outside of formal health care settings, with free-text responses indicating interest in such an approach to manage menopause.

Frequently searched for treatment side effects online included weight gain, mood changes, and risk of cancer. Concerns about cancer related to menopause treatments are frequent, fueled by conflicting research publications about the risks associated with HRT. However, for most individuals, the benefits of HRT outweigh any potential risks [27], with HRT conferring both short-term symptom relief and longer-term protective health benefits [28]. Fears of HRT may also be propagated by HCPs who reportedly infrequently provide an opportunity to discuss HRT, including both potential risks and benefits [29]. Therefore, the internet may offer an effective modality for providing comprehensive information about HRT, particularly with regard to the risks associated with it. Thematic analysis revealed a perception that misinformation is spread online about HRT, potentially contributing to unnecessary confusion or distress when seeking formal care.

The type of general advice most sought via online searches was how to ask for support, both generally and from an HCP. Evidence indicates poor formal care experiences for menopause [26], with a high level of self-advocacy required. Therefore, increasing the skills needed to ask for help and advocate for support and health care is clearly needed, with the internet offering a convenient source to deliver this information. However, it seems that it may not yet be fulfilling this need, with free-text responses requesting that online resources include more advice and recommendations related to this. In addition, thematic analysis revealed requests for how to ask for support in the workplace and workplace rights. Previous evidence indicates that generally workplaces do not provide information about menopause [30], potentially necessitating online searching. Similarly, thematic analysis also indicated a perception that information provision is lacking for workplaces. Given that menopause is associated with women leaving the workforce [5], it is imperative that workplaces are well informed and prepared to support employees, and as such, high-quality online resources for both employees and employers are required.

Finally, this study demonstrates the importance of social connection in menopause. Free-text data asked for online resources to provide directories of support groups. Participants also stated the utility of support groups in online spaces, such as social media, to share information and experiences. This is similar to previous qualitative research in postmenopausal women, which found that support groups, including online support groups, enable knowledge sharing and reduce feelings of isolation [31]. Internet-based social networks have also been demonstrated to be beneficial for connecting with others who have a similar experience, particularly for individuals whose experience may be considered “atypical” [8]. Similarly, participants also requested narrative accounts of menopause, stating that these types of passive peer support would achieve similar benefits. This finding reflects other works that also found that information is used to better understand and validate menopause symptoms and experiences [32].

### Strengths and Limitations

The findings from this study should be interpreted alongside several considerations. First, due to the characteristics of the study sample, extreme care must be taken when extrapolating these findings to the wider population. The participants in the study were disproportionately White (592/627, 95%), compared to the recorded national average of 81.7% in the 2021 census [33]. In addition, much of the sample endorsed a high educational achievement of at least an advanced-level certificate or equivalent. This is notable as education has a demonstrable influence on health literacy [34] and how one experiences and interacts with health care [35]. It is likely that different groups will differ in their use of and perspectives on online searching for menopause information, and the study’s findings will not reflect the perspectives from minority groups.

Second, there is a risk of selection bias due to the online recruitment method. Individuals who are familiar with using the internet, particularly those who possess the knowledge required to find health-related information, were more likely to see the study advertisements and choose to participate. This may have resulted in a sample that is not fully representative of the broader population. Individuals with limited internet access, lower digital literacy, or less experience with navigating online health resources will likely have been underrepresented. In addition, the recruitment source for individual participants was not tracked, and therefore, we are unable to analyze whether differences in recruitment pathways (eg, social media, professional networks, or word of mouth) may have influenced participant characteristics or responses. Therefore, we are unable to evaluate the risk of any potential recruitment bias. Consequently, a number of participants may have been recruited into this study after previously taking part in research conducted by the Cambridge Centre for Neuropsychiatric Research and indicating a willingness to be contacted for future studies. This may have introduced additional bias, as these individuals could differ from the general population in terms of research interest or health information–seeking behaviors.

Consequently, the findings may not reflect the use of the internet as an informational source for menopause in the wider population, with the behaviors and preferences of those who are less comfortable with or have limited access to digital tools potentially differing significantly from the findings presented. For example, previous work has demonstrated that higher educational attainment and digital literacy are associated with higher rates of digital health information seeking [36].

Third, while the survey used in this study was reviewed and approved by a psychiatrist and provided a broad overview of online search behavior and informational needs related to menopause, some key aspects may have been overlooked, as it was not reviewed by a wider group of relevant stakeholders. Although we attempted to mitigate this limitation by including free-text fields where participants could share additional insights, we acknowledge that some key data may still not have been captured. Future studies could benefit from a more robust co-design process during survey development to ensure relevant data capture.

Finally, because the free-text question followed the questions with prespecified answer options, the responses may have been influenced by them.

### Future Directions

Future research should aim to address the limitations in sample diversity present in this study. The overrepresentation of White, highly educated participants highlights the need for studies involving more ethnically and socioeconomically diverse populations to ensure findings are broadly applicable. Recruitment strategies that go beyond online platforms, as were used in this study, should also be explored to reduce selection bias and better capture the experiences of individuals who may have limited digital access or lower digital literacy.

Furthermore, given the apparent widespread reliance on the internet as a primary source of information about menopause, it is essential that research be undertaken to complete objective assessments of the quality and accuracy of these online resources. Such evaluations will help identify further gaps in information coverage or areas where resources could be improved and assess the risk of potential misinformation. This work is particularly important given the high levels of trust and perceived accuracy of online informational resources for menopause expressed by participants in this study.

Beyond research, this study also points to clear gaps in the available menopause-related information from the perspective of those seeking it and suggests opportunities to develop more tailored resources for underrepresented groups. Therefore, the current findings may be valuable for individuals or organizations involved in developing online or digital resources related to menopause information.

### Conclusions

This study found that self-directed online searches for information about menopause are frequent, commonly being the first or potentially the only source of information sought by some women. Online searches are conducted principally to find information related to menopause symptoms and treatments. The information available online is widely trusted and generally perceived as accurate, with the websites of recognized health care services being perceived as most trustworthy. However, qualitative data indicate that information is perceived as generic, and there are several risk factors associated with distrust in information, including a lack of informational source transparency and perceived deceptive advertising practices. There are also apparent differences in the online search needs and behaviors between different menopausal groups. Future resource development should consider the role of individual characteristics and preferences, including the specific stage of menopause.

The participants in this study have highlighted some specific problems with the currently available online information about menopause and provided suggestions of what they would like to see changed. In particular, participants requested a more comprehensive and detailed list of all menopause symptoms and clearer guidance available on treatment options, including nonformal symptom management options. Internet users also seem to seek practical advice related to self-management of symptoms and on how to effectively communicate with HCPs and employers. In addition, the study revealed the potential for the internet to provide social support, both passively (ie, through narrative accounts) and actively (ie, through support networks), with this being cited as beneficial for reducing uncertainty about the menopausal experience.

## Appendix

Multimedia Appendix 1 The Checklist for Reporting Results of Internet E-Surveys.

Multimedia Appendix 2 Survey questions.

Multimedia Appendix 3 Data tables.

## Abbreviations

GP: general practitioner
HCP: health care professional
HRT: hormone replacement therapy
NHS: National Health Service

## Data Availability

The datasets generated during and analyzed during this study are available from the corresponding author on reasonable request.
