# Supplementary material for: Understanding Experiences of and Unmet Needs in Online Searches for Menopause Information: An Exploratory Survey
Source: JMIR Form Res. 2025 Oct 1;9:e75335. doi: 10.2196/75335 (PMC12530155; doi:10.2196/75335)
Supplement: Multimedia Appendix 2 [file formative_v9i1e75335_app2.docx]

**Multimedia Appendix 2: Survey questions**

Start of Block: Demographics

What is your age?

________________________________________________________________

What gender do you identify with?

- Female (1)
- Male (2)
- Non-binary (3)
- Other (4)
- Prefer not to say (5)

What is your ethnic group?

- White (1)
- Black, Black British, Caribbean or African (2)
- Asian or Asian British (3)
- Arab (4)
- Mixed or multiple ethnic groups (5)

Which of the following best describes your relationship status?

- Single (1)
- Married/Civil partnership (2)
- Cohabiting (i.e., living with a partner without being married to or in a civil partnership with them) (3)
- Separated (4)
- Divorced (5)
- Other, please specify (6) __________________________________________________
- Prefer not to say (7)

| Page Break |  |
| --- | --- |

What is the highest level of education you have achieved?

- Below GCSE/equivalent (1)
- GCSE/Scottish higher/equivalent (2)
- A-Levels/International Baccalaureate (IB)/Advanced Higher (3)
- Undergraduate degree (e.g., BSc, BA) (4)
- Postgraduate degree or qualification (e.g., Masters, PhD, EdD, DClinPsy, PGCE) (5)
- Other, please specify (6) __________________________________________________
- Prefer not to answer (7)

What is your current employment status? Select all that apply.

- Employed full-time (1)
- Employed part-time (2)
- Self-employed (3)
- Parental leave or taking time off to care for a family member (4)
- Student (5)
- Retired (6)
- Voluntary work (7)
- Unemployed (8)
- ⊗Prefer not to answer (9)

What is your current annual **household** income before tax?

 This may also include your partner's/family's annual income before tax, if living together.

- Less than £15,000 (1)
- £15,001 - £25,000 (2)
- £25,001 - £35,000 (3)
- £35,001 - £45,000 (4)
- £45,001 - £55,000 (5)
- £55,001 - £65,000 (6)
- £65,001 - £75,000 (7)
- £75,001 - £85,000 (8)
- More than £85,000 (9)
- Prefer not to answer (10)

End of Block: Demographics

Start of Block: Factors affecting the menopause

Menstrual cycles change with age. **The menopause transition** (also known as perimenopause) can begin eight to 10 years before the menopause, when the ovaries gradually produce less oestrogen. **The menopause** is a stage in life when you stop having your monthly period for reasons other than pregnancy, breastfeeding, or hormonal contraception. Symptoms associated with the menopause/menopause transition can include hot flushes, mood changes, night sweats, and decreased sex drive.

 Which of the following options best describes you?

- Early perimenopausal (significant change in the length of your menstrual bleed or the time between periods that is not due to pregnancy/breastfeeding, stress, or a medical condition) (1)
- Late perimenopause (no menstrual bleeding in 3-11 months not due to pregnancy/breastfeeding, stress, or a medical condition) (2)
- Natural menopause (no menstrual bleeding in 12 months not due to pregnancy/breastfeeding, stress, or a medical condition) (3)
- Surgical menopause (no menstrual bleeding due to hysterectomy with one or two ovaries retained or other medical procedure) (4)

End of Block: Factors affecting the menopause

Start of Block: Why was the internet used

Have you used the internet as a source of information **about the menopause**?

- Yes (1)
- No (2)

Display This Question:

If Have you used the internet as a source of information about the menopause? = No

Why did you not use the internet to find information **about the menopause**? Select all that apply.

- Not interested in finding information about the menopause (1)
- Don't know where to start looking (2)
- Not confident using the internet to find this information (3)
- Don’t trust online sources (4)
- Already educated about the menopause (5)
- Have a good support network (6)
- Received good care from healthcare provider (7)
- Experienced few bothersome symptoms (8)
- Other, please specify (9) __________________________________________________

| Page Break |  |
| --- | --- |

Display This Question:

If Have you used the internet as a source of information about the menopause? = Yes

What type(s) of online source have you used? Select all that apply.

- NHS online / NHS Inform (Scotland) (1)
- Sites reporting other people's experienced; e.g. Healthtalk (2)
- Sites suggesting in-person support options; e.g. Menopause café (3)
- Charities providing educational materials for the public; e.g.Women's Health Concern, Menopause Matters, The Menopause Charity (4)
- Websites aimed at healthcare professionals (5)
- Social media (6)
- Scientific literature; e.g. ScienceDirect (7)
- Online news sites with reports about the menopause (8)
- Other, please specify (9) __________________________________________________

Display This Question:

If Have you used the internet as a source of information about the menopause? = Yes

How did you find the online sources? Select all that apply.

- Google search (1)
- Provided with specific suggestions from healthcare provider (2)
- Provided with specific suggestion from friends/family (3)
- Followed links from other sites/social media (4)
- Other, please specify (5) __________________________________________________

| Page Break |  |
| --- | --- |

To what extent do you feel that information found online about the **menopause** is accurate?

- Not at all accurate (1)
- Partially accurate (2)
- Mostly accurate (3)
- Completely accurate (4)
- Not sure (5)

To what extent do you trust information about the **menopause** found online?

- Not at all (1)
- Slightly (2)
- Moderately (3)
- Very much so (4)

| 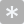 |
| --- |

What type of site would you trust the most? Select up to 3.

- Social media (1)
- Official NHS website (2)
- Charities providing information about the internet; e.g. The Menopause Society (3)
- Scientific literature found online; e.g. ScienceDirect (4)
- Websites of medical bodies; e.g. Royal College of Gynaecologists (5)
- Online news sites with reports about the menopause (6)
- Other, please specify (7) __________________________________________________

End of Block: Why was the internet used

Start of Block: Use of the internet

Was the internet your first choice for researching **the menopause**?

- Yes (1)
- No (2)

Did you use other sources to find information **about the menopause** as well as using the internet? This includes contacting a health care professional.

- Yes (1)
- No (2)

Display This Question:

If Was the internet your first choice for researching the menopause? = Yes

And Did you use other sources to find information about the menopause as well as using the internet?... = No

Why did you not use any sources in addition to using the internet? Select all that apply.

- Found enough information online (1)
- Don't trust healthcare professional (2)
- Not comfortable discussing the menopause with healthcare professional (3)
- Didn't feel it was worth contacting healthcare professional (4)
- Too embarrassed to talk to friends or family (5)
- Don't have anyone you're comfortable talking to about the menopause (6)
- Couldn't physically attend an appointment with a healthcare professional or was unable to book an appointment (7)
- Didn't think symptoms were severe enough (8)
- Other, please specify (9) __________________________________________________

Display This Question:

If Did you use other sources to find information about the menopause as well as using the internet?... = Yes

Which sources of information did you use in addition to the internet to find information **about the menopause**? Select all that apply.

- General practitioner (NHS GP) (1)
- Gynaecologist (2)
- Another consultant; a specialist doctor e.g. a psychiatrist (3)
- Practice nurse (4)
- Pharmacist (5)
- Family (6)
- Friends (7)
- Support group (8)
- Religious leader (9)
- Other, please specify (10) __________________________________________________

Display This Question:

If Did you use other sources to find information about the menopause as well as using the internet?... = Yes

Which sources of information did you feel provided the most useful information? Select all that apply.

- Online resources (1)

Display This Choice:

If Which sources of information did you use in addition to the internet to find information about th... = General practitioner (NHS GP)

- General practitioner (NHS GP) (2)

Display This Choice:

If Which sources of information did you use in addition to the internet to find information about th... = Gynaecologist

- Gynaecologist (3)

Display This Choice:

If Which sources of information did you use in addition to the internet to find information about th... = Another consultant; a specialist doctor e.g. a psychiatrist

- Another consultant; a specialist doctor e.g. a psychiatrist (4)

Display This Choice:

If Which sources of information did you use in addition to the internet to find information about th... = Practice nurse

- Practice nurse (5)

Display This Choice:

If Which sources of information did you use in addition to the internet to find information about th... = Pharmacist

- Pharmacist (6)

Display This Choice:

If Which sources of information did you use in addition to the internet to find information about th... = Family

- Family (7)

Display This Choice:

If Which sources of information did you use in addition to the internet to find information about th... = Friends

- Friends (8)

Display This Choice:

If Which sources of information did you use in addition to the internet to find information about th... = Support group

- Support group (9)

Display This Choice:

If Which sources of information did you use in addition to the internet to find information about th... = Religious leader

- Religious leader (10)
- Other, please specify (11) __________________________________________________

Display This Question:

If Was the internet your first choice for researching the menopause? = Yes

And Did you use other sources to find information about the menopause as well as using the internet?... = Yes

Why did you choose to use another source of information after using the internet? Select all that apply.

- Couldn't find the information you were looking for online (1)
- Wanted verification that information you found online was correct and could be believed (2)
- Overwhelming amount of information found online (3)
- Found unclear information online and wanted to clarify it with a healthcare professional (4)
- Wanted as much information as possible before making a decision, e.g. treatment options (5)
- Wanted to prepare for appointment with a healthcare professional (6)
- Used the information found online to signpost you towards other sources of help (7)
- Wanted to get help from a healthcare professional as a result of finding information online (8)
- Other, please specify (9) __________________________________________________

Display This Question:

If Was the internet your first choice for researching the menopause? = No

And Did you use other sources to find information about the menopause as well as using the internet?... = Yes

Why did you use the internet after consulting another source of information? Select all that apply.

- Healthcare professional signposted you to a site for information (1)
- Healthcare professional unwilling to prescribe treatment for symptoms of the menopause (2)
- Referral to a specialist was refused by healthcare professional (3)
- Felt dismissed by healthcare professional (4)
- To access resources recommended by friends or family (5)
- Didn't feel enough information had been provided otherwise (6)
- Didn't feel that the information provided had been useful (7)
- Wanted to collect as much information as possible (8)
- Other, please specify (9) __________________________________________________

End of Block: Use of the internet

Start of Block: What information was searched for

What information **related to the menopause** were you searching for? Select all that apply.

- Information about what causes the menopause (1)
- Menopause treatment side-effects (2)
- Health risks related to the menopause (3)
- Information about pregnancy and the menopause (4)
- Consequences of the menopause (e.g. difficulties with ...) (5)
- Menopause symptoms (6)
- Menopause treatment options (7)
- Private care options for the menopause (8)
- Support groups for the menopause (9)
- Information about surgical menopause (10)
- General advice (i.e. how to ...) (11)
- Self-help tips or strategies (e.g. exercise, specific diets for the menopause, mindfulness or other stress reduction activities) (12)
- Other, please specify (13) __________________________________________________

Display This Question:

If What information related to the menopause were you searching for? Select all that apply. = Menopause treatment side-effects

Which specific side-effects were you researching? Select all that apply.

- Risk of cancer (e.g. ovarian, breast, cervical, uterine) (1)
- Breast tenderness (2)
- Irregular bleeding (3)
- Nausea (4)
- Headaches (5)
- Mood changes (6)
- Risk of blood clots (7)
- Risk of heart disease (8)
- Dizziness (9)
- Decreased interest in sex (10)
- Weight gain (11)
- Other, please specify (12) __________________________________________________

Display This Question:

If What information related to the menopause were you searching for? Select all that apply. = Health risks related to the menopause

Which specific health risks were you interested in? Select all that apply.

- Heart disease (1)
- Stroke (2)
- Osteoporosis (weakening bones) (3)
- Urinary tract infections (UTIs) (4)
- Diabetes (5)
- Dementia (6)
- Alzheimer's (7)
- Blood pressure changes (8)
- Other, please specify (9) __________________________________________________

Display This Question:

If What information related to the menopause were you searching for? Select all that apply. = Menopause symptoms

Which symptoms were you researching? Select all that apply. Please be aware that your responses to this survey are completely anonymous. Giving us your email is done separately and we will not know who has completed which survey. This means that we cannot get back to you about particular things that you have told us. If you feel that you are struggling with your mental health please contact your GP. If you need to talk at any time of the day or night to confidential and trained listeners, call the Samaritans by dialling 116 123. In an urgent crisis call the NHS mental health line by dialling 111.

- General symptoms - didn't search for any in particular (1)
- Hot flushes (2)
- Night sweats (3)
- Heart palpitations (e.g. feeling that your heart is racing, irregular, or beating hard in your chest) (4)
- Feeling tense or nervous (5)
- Difficulty sleeping not due to night sweats (6)
- Feeling excitable (7)
- Anxiety or panic attacks (8)
- Difficulty concentrating (9)
- Feeling tired, or that you have no energy (10)
- Loss of interest in most things (11)
- Feeling unhappy or depressed (12)
- Crying spells (13)
- Irritability (14)
- Feeling dizzy or faint (15)
- Pressure or tightness in your head (16)
- Headaches (17)
- Parts of your body feeling numb (18)
- Muscle or joint aches or pains (19)
- Loss of feeling in hands or feet (20)
- Breathing difficulties (21)
- Change in your sexual desire (22)
- Mood swings (23)
- Poor memory (24)
- Brain fog (25)
- Difficulties in multi-tasking (26)
- Recurrent urinary tract infections (UTIs) (27)
- Frequent urination or involuntary urination when laughing or coughing (28)
- Vaginal dryness (29)
- Vaginal irritation, burning, or itching (30)
- Irregular periods (31)
- Change in skin texture or appearance (32)
- Hair loss/thinning (33)
- Growth of facial hair (34)
- Tinnitus (e.g. hearing a ringing or buzzing inside your head) (35)
- Loss of self-esteem (36)
- Loss of confidence (37)
- Painful sex (38)
- Reduced feelings of intimacy (39)
- Feelings of wanting to die, or that life is not worth living (40)
- Feelings of hopelessness (41)

Display This Question:

If What information related to the menopause were you searching for? Select all that apply. = Menopause treatment options

Which treatments or support options were you searching for? Select all that apply.

- Oral HRT (1)
- Transdermal HRT (skin patches, gel, spray) (2)
- Vaginal HRT (vaginal cream, gel, pessary, tablet or ring) (3)
- Intrauterine system (IUS or Mirena coil) (4)
- Testosterone (5)
- Antidepressants (e.g. citalopram, fluoxetine, sertraline, duloxetine) (6)
- Blood pressure medication for the menopause (e.g. clonidine) (7)
- Epilepsy or seizure medication (e.g. gabapentin) (8)
- Non-medicated lubricant (9)
- Non-prescription medication or supplements (10)
- Complementary therapy (e.g. acupuncture) (11)
- Lifestyle changes (12)
- Counselling, CBT or other talking therapy for symptoms such as anxiety and depression related to the menopause (13)
- Other, please specify (14) __________________________________________________

Display This Question:

If What information related to the menopause were you searching for? Select all that apply. = General advice (i.e. how to ...)

What kind of advice were you looking for? Select all that apply.

- How to talk to your partner about the menopause (1)
- How to talk about the menopause to other family members or friends (2)
- How to discuss the menopause and its effects to your employer (3)
- How to ask for support (4)
- How to talk to your children about the menopause (5)
- How to talk to your healthcare professional about the menopause (6)
- Other, please specify (7) __________________________________________________

End of Block: What information was searched for

Start of Block: Additional comments

Did you find what you were looking for **online**?

- Yes (1)
- Some but not all (3)
- No (2)

Is there anything that you would like to see in **online resources** about the menopause that is currently not available?

________________________________________________________________

________________________________________________________________

________________________________________________________________

________________________________________________________________

________________________________________________________________

End of Block: Additional comments
