# Supplementary material for: Understanding Experiences of and Unmet Needs in Online Searches for Menopause Information: An Exploratory Survey
Source: JMIR Form Res. 2025 Oct 1;9:e75335. doi: 10.2196/75335 (PMC12530155; doi:10.2196/75335)
Supplement: Multimedia Appendix 3 [file formative_v9i1e75335_app3.docx]

**Multimedia Appendix 3.** Data tables (Multimedia appendix Table S1-S13)

| **Table S1.** Sociodemographic information | | | | |  | |  | |  | |
| --- | --- | --- | --- | --- | --- | --- | --- | --- | --- | --- |
|  | **Overall (N=627, 100.00%)** | | **Early perimenopause (n=171, 27.27%)** | | **Late perimenopause (n=125, 19.94%)** | | **Natural menopause (n=262, 41.79%)** | | **Surgical/**  **medically induced menopause**  **(n=69, 11.00%)** | |
|  | **n (%)** | **Mean (SD; range)** | **n (%)** | **Mean (SD; range)** | **n (%)** | **Mean (SD; range)** | **n (%)** | **Mean (SD; range)** | **n (%)** | **Mean (SD; range)** |
| **Socio-demographics** | | | | | | | | | | |
| Age |  | 52.40 (4.68; 31-71) |  | 48.82 (3.48; 40-58) |  | 51.98 (3.58; 42-61) |  | 55.00 (3.64; 45-71) |  | 52.20 (6.07; 31-64) |
| **Gender** | | | | | | | | | | |
| Female | 617 (98.41) |  | 165 (96.49) |  | 124 (99.20) |  | 259 (98.85) |  | 69 (100.00) |  |
| Male | 2 (0.32) |  | 1 (0.58) |  | 1 (0.80) |  | 0 |  | 0 |  |
| Non-binary | 1 (0.16) |  | 1 (0.58) |  | 0 |  | 0 |  | 0 |  |
| Other | 5 (0.80) |  | 3 (1.75) |  | 0 |  | 2 (0.76) |  | 0 |  |
| Prefer not to answer | 2 (0.32) |  | 1 (0.58) |  | 0 |  | 1 (0.38) |  | 0 |  |
| **Ethnicity** | | | | | | | | | | |
| White | 592 (94.74) |  | 160 (95.57) |  | 120 (96.00) |  | 250 (95.42) |  | 64 (92.75) |  |
| Black, Black British, Caribbean or African | 16 (2.55) |  | 4 (2.34) |  | 3 (2.40) |  | 5 (1.91) |  | 4 (5.80) |  |
| Asian or Asian British | 14 (2.23) |  | 7 (4.09) |  | 2 (1.60) |  | 5 (1.91) |  | 0 |  |
| Mixed or multiple ethnic groups | 3 (0.48) |  | 0 |  | 0 |  | 2 (0.76) |  | 1 (1.45) |  |
| **Marital status** | | | | | | | | | | |
| Single | 86 (13.72) |  | 23 (13.45) |  | 17 (13.60) |  | 40 (15.27) |  | 6 (8.70) |  |
| Married or civil partnership | 379 (60.45) |  | 111 (64.91) |  | 81 (64.80) |  | 143 (54.58) |  | 44 (63.77) |  |
| Cohabiting | 74 (11.80) |  | 17 (9.94) |  | 14 (11.20) |  | 33 (12.60) |  | 10 (14.49) |  |
| Separated | 23 (3.67) |  | 6 (3.51) |  | 2 (1.60) |  | 15 (5.73) |  | 0 |  |
| Divorced | 41 (6.54) |  | 8 (4.68) |  | 5 (4.00) |  | 23 (8.78) |  | 5 (7.25) |  |
| Other | 18 (2.87) |  | 3 (1.75) |  | 4 (3.20) |  | 8 (3.05) |  | 3 (4.35) |  |
| Prefer not to answer | 6 (0.96) |  | 3 (1.75) |  | 2 (1.60) |  | 0 |  | 1 (1.45) |  |
| **Education** | | | | | | | | | | |
| Below GCSE or equivalent | 37 (5.90) |  | 6 (3.51) |  | 6 (4.80) |  | 19 (7.25) |  | 6 (8.70) |  |
| GCSEs, Scottish Higher or equivalent | 106 (16.91) |  | 15 (8.77) |  | 25 (20.00) |  | 55 (20.99) |  | 11 (15.94)17 (24.64) |  |
| A-Levels, International Baccalaureate (IB) or equivalent | 133 (21.21) |  | 34 (19.88) |  | 30 (24.00) |  | 52 (19.85) |  | 17 (24.64) |  |
| Undergraduate degree | 152 (24.24) |  | 46 (26.90) |  | 32 (25.60) |  | 56 (21.37) |  | 18 (26.09) |  |
| Postgraduate degree | 154 (24.56) |  | 58 (33.92) |  | 27 (21.60) |  | 56 (21.37) |  | 13 (18.84) |  |
| Other | 26 (4.15) |  | 10 (5.85) |  | 1 (0.80) |  | 12 (4.58) |  | 3 (4.35) |  |
| Prefer not to answer | 19 (3.03) |  | 2 (1.17) |  | 4 (3.20) |  | 12 (4.58) |  | 1 (1.45) |  |
| **Employment*** | | | | | | | | | | |
| Employed full-time | 289 (46.09) |  | 83 (48.45) |  | 64 (51.20) |  | 113 (43.13) |  | 29 (42.03) |  |
| Employed part-time | 149 (23.76) |  | 44 (25.73) |  | 26 (20.80) |  | 65 (24.81) |  | 14 (20.29) |  |
| Self-employed | 71 (11.32) |  | 24 (14.04) |  | 16 (12.80) |  | 26 (9.92) |  | 5 (7.25) |  |
| Parental leave | 26 (4.15) |  | 8 (4.68) |  | 3 (2.40) |  | 9 (3.44) |  | 6 (8.70) |  |
| Student | 4 (0.64) |  | 2 (1.17) |  | 0 |  | 1 (0.38) |  | 1 (1.45) |  |
| Retired | 39 (6.22) |  | 1 (0.58) |  | 4 (3.20) |  | 27 (10.31) |  | 7 (10.14) |  |
| Volunteer | 14 (2.23) |  | 5 (2.92) |  | 2 (1.60) |  | 6 (2.29) |  | 1 (1.45) |  |
| Unemployed | 44 (7.02) |  | 9 (5.26) |  | 11 (8.80) |  | 17 (6.49) |  | 7 (10.14) |  |
| Prefer not to answer | 11 (1.75) |  | 2 (1.17) |  | 3 (2.40) |  | 3 (1.15) |  | 3 (4.35) |  |
| **Annual household income (before tax)** | | | | | | | | | | |
| Less than £15,000 | 49 (7.81) |  | 10 (5.85) |  | 8 (6.40) |  | 28 (10.69) |  | 3 (4.35) |  |
| £15,001-£25,000 | 69 (11.00) |  | 19 (11.11) |  | 9 (7.20) |  | 36 (13.74) |  | 5 (7.25) |  |
| £25,001-£35,000 | 73 (11.64) |  | 15 (8.77) |  | 18 (14.40) |  | 32 (12.21) |  | 8 (11.59) |  |
| £35,001-£45,000 | 73 (11.64) |  | 21 (12.28) |  | 12 (9.60) |  | 29 (11.07) |  | 11 (15.94) |  |
| £45,001-£55,000 | 69 (11.00) |  | 16 (9.36) |  | 14 (11.20) |  | 32 (12.21) |  | 7 (10.14) |  |
| £55,001-£65,000 | 49 (7.81) |  | 16 (9.36) |  | 12 (9.60) |  | 19 (7.25) |  | 2 (2.90) |  |
| £65,001-£75,000 | 37 (5.90) |  | 15 (8.77) |  | 7 (5.60) |  | 13 (4.96) |  | 2 (2.90) |  |
| £75,0001-£85,000 | 33 (5.26) |  | 10 (5.85) |  | 9 (7.20) |  | 10 (3.82) |  | 4 (5.80) |  |
| More than £85,001 | 78 (12.44) |  | 27 (15.79) |  | 15 (12.00) |  | 25 (9.54) |  | 11 (15.94) |  |
| Prefer not to answer | 97 (15.47) |  | 22 (12.87) |  | 21 (16.80) |  | 38 (14.50) |  | 16 (23.19) |  |

Key. * Total percentage may exceed 100% as participants were able to select more than one type of employment. GCSE: General Certificate of Secondary Education; A-Level: Advanced Level

**Table S2.** Reasons for not using the internet as a source of information

|  | **Overall (n=46)** | **Early perimenopause (n=9)** | **Late perimenopause (n=13)** | **Natural menopause (n=20)** | **Surgical/**  **medically induced menopause**  **(n=4)** |
| --- | --- | --- | --- | --- | --- |
| **Why did you not use the internet to find information about the menopause** | | | | | |
| Not interested in finding information about the menopause | 3 (6.52) | 0 | 1 (7.69) | 2 (10.00) | 0 |
| Don’t know where to start looking | 17 (39.96) | 3 (33.33) | 8 (61.54) | 6 (30.00) | 0 |
| Not confident using the internet to find this information | 6 (13.04) | 1 (11.11) | 1 (7.69) | 3 (15.00) | 1 (5.00) |
| Don’t trust online sources | 6 (13.04) | 1 (11.11) | 0 | 4 (20.00) | 1 (5.00) |
| Already educated about the menopause | 8 (17.39) | 0 | 2 (15.38) | 5 (25.00) | 1 (5.00) |
| Have a good support network | 3 (6.52) | 1 (1.11) | 0 | 2 (10.00) | 0 |
| Received good care from HCP | 6 (13.04) | 2 (2.22) | 3 (23.08) | 1 (5.00) | 0 |
| Experiences few bothersome symptoms | 9 (19.57) | 3 (3.33) | 1 (7.69) | 5 (25.00) | 0 |
| Other | 5 (10.87) | 1 (1.11) | 0 | 2 (10.00) | 2 (10.00) |

Key. HCP: Health care professional

**Table S3.** Use of the internet for information searching

|  | **Overall (n=581)** | **Early perimenopause (n=162)** | **Late perimenopause (n=112)** | **Post-menopause (n=242)** | **Surgical/**  **medically induced menopause**  **(n=65)** |
| --- | --- | --- | --- | --- | --- |
| **How did you find the online sources?** | | | | | |
| Google Search | 511 (87.95) | 144 (88.89) | 91 (81.25) | 216 (89.26) | 60 (92.31) |
| Provided with specific suggestions from HCP | 96 (16.52) | 28 (17.28) | 21 (18.75) | 33 (13.64) | 14 (21.54) |
| Provided with specific suggestions from friends/family | 121 (20.83) | 42 (25.93) | 27 (24.11) | 43 (17.77) | 9 (13.85) |
| Followed links from other sites/social media | 271 (46.64) | 77 (47.53) | 55 (49.11) | 108 (44.63) | 31 (47.69) |
| Other | 22 (3.79) | 6 (3.70) | 5 (4.46) | 10 (4.13) | 1 (1.54) |
| **Was the internet your first choice for researching the menopause?** | | | | | |
| Yes | 489 (84.17) | 144 (88.89) | 88 (78.57) | 204 (84.30) | 53 (81.54) |
| No | 92 (15.83) | 18 (11.11) | 24 (21.43) | 38 (15.70) | 12 (18.46) |
| **Did you use other sources to find information about the menopause as well as using the internet? This includes contacting a health care professional.** | | | | | |
| Yes | 439 (75.56) | 126 (77.78) | 88 (78.57) | 174 (71.90) | 51 (78.46) |
| No | 142 (24.44) | 36 (22.22) | 24 (21.43) | 68 (28.10) | 14 (21.54) |
| **Only consulted online sources** | 136 (23.41) | 34 (20.99) | 23 (20.54) | 65 (26.86) | 14 (21.54) |
| **Internet was the first choice, but another source was also consulted** | 353 (60.75) | 110 (67.90) | 65 (58.04) | 139 (57.44) | 39 (60.00) |
| **Internet consulted after another source** | 86 (14.80) | 16 (9.88) | 23 (20.54) | 35 (14.46) | 12 (18.46) |
| **Did you find what you were looking for online?** | | | | | |
| Yes | 176 (30.29) | 52 (32.10) | 33 (29.46) | 75 (30.99) | 16 (24.62) |
| Some but not all | 379 (65.23) | 102 (62.96) | 74 (66.07) | 157 (64.88) | 46 (70.77) |
| No | 26 (4.48) | 8 (4.94) | 5 (4.46) | 10 (4.13) | 3 (4.62) |

Key. HCP: Health care professional

**Table S4.** Reason for using another source of information after using the internet

|  | **Overall (n=353)** | **Early perimenopause (n=110)** | **Late perimenopause (n=65)** | **Post-menopause (n=139)** | **Surgical/**  **medically induced menopause**  **(n=39)** |
| --- | --- | --- | --- | --- | --- |
| **Why did you choose to use another source of information after using the internet?** | | | | | |
| Couldn’t find the information you were looking for online | 48 (13.60) | 8 (7.27) | 12 (18.46) | 19 (13.67) | 9 (23.08) |
| Wanted verification that information you found online was correct and could be believed | 164 (46.46) | 48 (43.64) | 40 (61.54) | 55 (39.57) | 21 (53.85) |
| Overwhelming amount of information found online | 110 (31.16) | 45 (40.91) | 21 (32.31) | 34 (24.46) | 10 (25.64) |
| Found unclear information online and wanted to clarify it with a health care professional | 79 (22.38) | 25 (22.73) | 18 (27.69) | 27 (19.42) | 9 (23.08) |
| Wanted as much information as possible before making a decision (e.g., treatment options) | 183 (51.84) | 52 (47.27) | 39 (60.00) | 71 (51.08) | 21 (53.85) |
| Wanted to prepare for appointment with a HCP | 120 (33.99) | 41 (37.27) | 22 (33.85) | 44 (31.65) | 13 (33.33) |
| Used the information found online to signpost you towards other sources of help | 75 (21.25) | 22 (20.00) | 14 (21.54) | 30 (21.58) | 9 (23.08) |
| Wanted to get help from a HCP as a result of finding information online | 132 (37.39) | 45 (40.91) | 21 (32.31) | 48 (34.58) | 18 (46.15) |
| Other | 19 (5.38) | 5 (4.55) | 5 (7.69) | 6 (4.32) | 3 (7.69) |

Key. HCP: Health care professional

**Table S5.** Reason for not using other information sources in addition to the internet

|  | **Overall (n=136)** | **Early perimenopause (n=34)** | **Late perimenopause (n=23)** | **Post-menopause (n=65)** | **Surgical/**  **medically induced menopause**  **(n=14)** |
| --- | --- | --- | --- | --- | --- |
| **Why did you not use any sources in addition to using the internet?** | | | | | |
| Found enough information online | 55 (40.44) | 13 (9.56) | 7 (30.43) | 31 (47.69) | 4 (28.57) |
| Don’t trust HCP | 8 (5.88) | 4 (2.94) | 0 | 1 (1.54) | 3 (21.43) |
| Not comfortable discussing the menopause with HCP | 9 (6.62) | 2 (1.47) | 2 (8.70) | 4 (6.15) | 1 (7.14) |
| Didn’t feel it was worth contacting HCP | 57 (41.91) | 14 (10.29) | 10 (43.48) | 28 (43.08) | 5 (35.71) |
| Too embarrassed to talk to friends or family | 10 (7.35) | 2 (1.47) | 3 (13.04) | 4 (6.15) | 1 (7.14) |
| Don’t have anyone you’re comfortable talking to about the menopause | 16 (11.76) | 4 (2.94) | 5 (21.74) | 5 (7.69) | 2 (14.29) |
| Couldn’t physically attend an appointment with a HCP or was unable to book an appointment | 32 (23.53) | 10 (7.35) | 8 (34.78) | 12 (18.46) | 2 (14.29) |
| Didn’t think symptoms were severe enough | 57 (49.91) | 16 (11.76) | 8 (34.78) | 29 (44.62) | 4 (28.57) |
| Other | 26 (19.12) | 6 (4.41) | 3 (13.04) | 12 (18.46) | 5 (35.71) |

Key. HCP: Health care professional

**Table S6.** Additional information sources accessed in addition to the internet

|  | **Overall (n=439)** | **Early perimenopause (n=126)** | **Late perimenopause (n=88)** | **Post-menopause (n=174)** | **Surgical/**  **medically induced menopause**  **(n=53)** |
| --- | --- | --- | --- | --- | --- |
| **Which sources of information did you use in addition to the internet to find information about the menopause?** | | | | | |
| NHS GP | 353 (80.41) | 103 (81.75) | 67 (76.14) | 31 (52.30) | 41 (80.39) |
| Gynecologist | 75 (17.08) | 18 (14.29) | 9 (10.23) | 21 (12.07) | 22 (43.14) |
| Another consultant; a specialist doctor (e.g., a psychiatrist) | 32 (7.29) | 6 (4.76) | 7 (7.95) | 8 (4.60) | 5 (9.80) |
| Practice nurse | 100 (22.78) | 30 (23.81) | 24 (27.27) | 26 (14.94) | 13 (25.49) |
| Pharmacist | 32 (7.29) | 2 (1.59) | 8 (9.09) | 10 (5.75) | 7 (13.73) |
| Family | 116 (26.44) | 37 (29.37) | 26 (29.55) | 27 (15.52) | 10 (19.61) |
| Friends | 220 (50.11) | 69 (54.76) | 46 (52.27) | 56 (32.18) | 20 (39.22) |
| Support group | 58 (13.21) | 13 (10.32) | 13 (14.77) | 13 (7.47) | 13 (25.49) |
| Religious leader | 0 | 0 | 0 | 0 | 0 |
| Other | 66 (15.03) | 16 (12.70) | 15 (17.05) | 21 (12.07) | 5 (9.80) |

Key. NHS: National Health Service; HCP: Health care professional

**Table S7.** Information about the menopause searched for online

|  | **Overall (n=581)** | **Early perimenopause (n=162)** | **Late perimenopause (n=112)** | **Post-menopause (n=242)** | **Surgical/**  **medically induced menopause**  **(n=)** |
| --- | --- | --- | --- | --- | --- |
| **What information related to the menopause were you searching for?** | | | | | |
| Information about what causes the menopause | 69 (11.88) | 22 (13.58) | 16 (14.29) | 24 (9.92) | 7 (10.77) |
| Menopause treatment side-effects | 312 (53.70) | 93 (57.41) | 58 (51.79) | 130 (53.72) | 31 (47.69) |
| Health risks related to the menopause | 275 (47.33) | 80 (49.38) | 56 (50.00) | 103 (42.56) | 36 (55.38) |
| Information about pregnancy and the menopause | 16 (2.75) | 7 (4.32) | 6 (5.36) | 3 (1.24) | 0 |
| Consequences of the menopause (e.g. difficulties with…) | 283 (48.71) | 78 (48.15) | 57 (50.89) | 114 (47.11) | 34 (52.31) |
| Menopause symptoms | 479 (82.44) | 139 (85.80) | 95 (84.82) | 195 (80.58) | 50 (76.92) |
| Menopause treatment options | 442 (76.08) | 132 (81.48) | 89 (79.46) | 174 (71.90) | 47 (72.31) |
| Private care options for the menopause | 65 (11.19) | 17 (10.49) | 21 (18.75) | 21 (8.68) | 6 (9.23) |
| Support groups for the menopause | 117 (20.14) | 30 (18.52) | 23 (20.54) | 49 (20.25) | 15 (23.08) |
| Information about surgical menopause | 33 (5.68) | 2 (1.23) | 1 (0.89) | 2 (0.83) | 28 (43.08) |
| General advice (i.e. how to…) | 155 (26.68) | 39 (24.07) | 37 (33.04) | 70 (28.93) | 9 (13.85) |
| Self-help tips or strategies (e.g. exercise, specific diets for the menopause, mindfulness or other stress reduction activities) | 318 (54.73) | 88 (54.32) | 67 (59.82) | 135 (55.79) | 28 (43.08) |
| Other, please specify | 32 (5.51) | 9 (5.56) | 8 (7.14) | 9 (3.72) | 6 (9.23) |

**Table S8.** Information about menopause-related health risks searched for online

|  | **Overall (n=275)** | | **Early perimenopause (n=80)** | | **Late perimenopause (n=56)** | | **Post-menopause (n=103)** | | **Surgical/**  **medically induced menopause**  **(n=36)** | |
| --- | --- | --- | --- | --- | --- | --- | --- | --- | --- | --- |
| **Which specific health risks were you interested in?** | | | | | | | | | | |
| Heart disease | | 124 (45.09) | | 31 (38.75) | | 26 (46.43) | | 45 (43.69) | | 22 (61.11) |
| Stroke | | 102 (37.09) | | 24 (30.00) | | 24 (44.64) | | 37 (35.92) | | 16 (44.44) |
| Osteoporosis | | 188 (68.36) | | 52 (65.00) | | 31 (55.36) | | 73 (70.87) | | 32 (88.89) |
| Urinary tract infections | | 79 (28.73) | | 17 (21.25) | | 26 (46.43) | | 23 (22.33) | | 13 (36.11) |
| Diabetes | | 51 (18.55) | | 11 (13.75) | | 11 (19.64) | | 21 (20.39) | | 8 (22.22) |
| Dementia | | 104 (37.82) | | 30 (37.50) | | 19 (26.79) | | 39 (37.86) | | 16 (44.44) |
| Alzheimer’s | | 75 (27.27) | | 21 (26.25) | | 15 (26.79) | | 28 (27.18) | | 11 (30.56) |
| Blood pressure changes | | 105 (38.18) | | 26 (32.50) | | 29 (51.79) | | 39 (37.86) | | 11 (30.56) |
| Other | | 39 (14.18) | | 14 (17.50) | | 6 (10.71) | | 14 (13.59) | | 5 (13.89) |

**Table S9.** Information about menopause treatment options searched for online

|  | **Overall (n=442)** | | **Early perimenopause (n=132)** | | **Late perimenopause (n=89)** | | **Post-menopause (n=174)** | | **Surgical/**  **medically induced menopause**  **(n=47)** | |
| --- | --- | --- | --- | --- | --- | --- | --- | --- | --- | --- |
| **Which treatments or support options were you searching for?** | | | | | | | | | | |
| Oral HRT | | 183 (41.20) | | 52 (139.39) | | 37 (41.57) | | 77 (44.25) | | 17 (9.77) |
| Transdermal HRT (skin patches, gel, spray) | | 289 (65.38) | | 89 (67.42) | | 59 (66.29) | | 114 (65.52) | | 27 (15.52) |
| Vaginal HRT (vaginal cream, gel, pessary, tablet or ring) | | 118 (26.70) | | 28 (21.21) | | 23 (25.84) | | 54 (31.03) | | 13 (7.47) |
| Intrauterine system (IUS or Mirena coil) | | 63 (14.25) | | 23 (17.42) | | 14 (15.73) | | 23 (13.22) | | 3 (1.72) |
| Testosterone | | 108 (24.43) | | 34 (25.76) | | 21 (23.60) | | 42 (24.14) | | 11 (6.32) |
| Antidepressants (e.g. citalopram, fluoxetine, sertraline, duloxetine) | | 68 (15.38) | | 21 (15.91) | | 16 (17.98) | | 22 (12.64) | | 9 (5.17) |
| Blood pressure medication for the menopause (e.g. clonidine) | | 29 (6.56) | | 8 (6.06) | | 5 (5.62) | | 12 (6.90) | | 4 (2.30) |
| Epilepsy or seizure medication (e.g. gabapentin) | | 6 (1.36) | | 2 (1.52) | | 2 (2.25) | | 1 (0.57) | | 1 (0.57) |
| Non-medicated lubricant | | 53 (11.99) | | 13 (9.85) | | 7 (7.87) | | 18 (10.34) | | 15 (8.62) |
| Non-prescription medication or supplements | | 139 (31.45) | | 40 (30.30) | | 23 (25.84) | | 62 (35.63) | | 14 (8.05) |
| Complementary therapy (e.g. acupuncture) | | 92 (20.81) | | 18 (13.64) | | 30 (33.71) | | 34 (19.54) | | 10 (5.75) |
| Lifestyle changes | | 171 (38.69) | | 55 (41.67) | | 35 (39.33) | | 64 (26.78) | | 17 (9.77) |
| Counselling, CBT, or other talking therapy for symptoms such as anxiety and depression related to the menopause | | 78 (17.65) | | 25 (18.94) | | 20 (22.47) | | 27 (15.52) | | 6 (3.45) |
| Other | | 33 (7.47) | | 7 (5.30) | | 12 (13.48) | | 8 (4.60) | | 6 (3.45) |

Key. HRT: Hormone replacement therapy; CBT: Cognitive behavioral therapy

**Table S10.** Information about treatment side-effects searched for online

|  | **Overall (n=312)** | | **Early perimenopause (n=93)** | | **Late perimenopause (n=58)** | | **Post-menopause (n=130)** | | **Surgical/**  **medically induced menopause**  **(n=31)** | |
| --- | --- | --- | --- | --- | --- | --- | --- | --- | --- | --- |
| **Which specific side-effects were you researching?** | | | | | | | | | | |
| Risk of cancer (e.g. ovarian, breast, cervical, uterine) | | 173 (55.45) | | 44 (47.31) | | 32 (55.17) | | 76 (58.46) | | 21 (67.74) |
| Breast tenderness | | 60 (19.23) | | 14 (15.05) | | 12 (20.69) | | 26 (20.00) | | 8 (25.81) |
| Irregular bleeding | | 76 (24.36) | | 31 (33.33) | | 21 (36.21) | | 22 (16.92) | | 2 (6.45) |
| Nausea | | 37 (11.86) | | 15 (16.13) | | 7 (12.07) | | 12 (9.23) | | 3 (9.68) |
| Headaches | | 120 (38.46) | | 34 (36.56) | | 20 (34.48) | | 54 (41.54) | | 12 (38.71) |
| Mood changes | | 178 (57.05) | | 53 (56.99) | | 34 (58.62) | | 72 (55.38) | | 19 (61.29) |
| Risk of blood clots | | 91 (29.17) | | 26 (27.96) | | 18 (31.03) | | 36 (27.69) | | 11 (35.48) |
| Risk of heart disease | | 77 (24.68) | | 21 (22.58) | | 15 (25.86) | | 33 (25.38) | | 8 (25.81) |
| Dizziness | | 66 (21.15) | | 26 (27.96) | | 19 (32.76) | | 18 (13.85) | | 3 (9.68) |
| Decreased interest in sex | | 110 (35.26) | | 26 (27.96) | | 25 (43.10) | | 46 (35.58) | | 13 (41.94) |
| Weight gain | | 187 (59.94) | | 47 (50.54) | | 38 (65.52) | | 84 (64.62) | | 18 (58.06) |
| Other | | 35 (11.22) | | 7 (7.53) | | 7 (12.07) | | 15 (11.54) | | 6 (19.35) |

**Table S11.** Specific advice for the menopause searched for online

|  | **Overall (n=155)** | | **Early perimenopause (n=39)** | | **Late perimenopause (n=37)** | | **Post-menopause (n=70)** | | **Surgical/**  **medically induced menopause**  **(n=9)** | |
| --- | --- | --- | --- | --- | --- | --- | --- | --- | --- | --- |
| **Which kind of advice were you looking for?** | | | | | | | | | | |
| How to talk to your partner about the menopause | | 19 (12.26) | | 5 (12.82) | | 5 (13.51) | | 7 (10.00) | | 2 (22.22) |
| How to talk about the menopause to other family members or friends | | 17 (10.97) | | 3 (7.69) | | 4 (10.81) | | 10 (14.29) | | 0 |
| How to discuss the menopause and its effects to your employer | | 22 (14.19) | | 2 (5.13) | | 9 (24.32) | | 10 (14.29) | | 1 (11.11) |
| How to ask for support | | 66 (42.58) | | 14 (35.90) | | 20 (54.05) | | 27 (38.57) | | 5 (55.56) |
| How to talk to your children about the menopause | | 8 (5.16) | | 2 (5.13) | | 4 (10.81) | | 2 (2.86) | | 0 |
| How to talk to your HCP about the menopause | | 65 (41.94) | | 16 (41.03) | | 15 (40.54) | | 30 (42.86) | | 4 (44.44) |
| Other | | 47 (30.32) | | 14 (35.90) | | 10 (27.03) | | 20 (28.57) | | 3 (33.33) |

Key. HCP: Health care professional

**Table S12.** Perceived quality of online information related to the menopause

|  | **Overall (n=627)** | **Early perimenopause (n=171)** | **Late perimenopause (n=125)** | **Post-menopause (n=262)** | **Surgical/**  **medically induced menopause**  **(n=69)** |
| --- | --- | --- | --- | --- | --- |
| **Perceived accuracy of information found online** | | | | | |
| Not at all accurate | 9 (1.44) | 2 (1.17) | 2 (1.60) | 4 (1.53) | 1 (1.45) |
| Partially accurate | 230 (36.68) | 62 (36.26) | 50 (40.00) | 83 (31.68) | 35 (50.72) |
| Mostly accurate | 304 (48.48) | 88 (51.46) | 60 (48.00) | 131 (50.00) | 25 (36.23) |
| Completely accurate | 21 (3.35) | 4 (2.34) | 2 (1.60) | 13 (4.96) | 2 (2.90) |
| Not sure | 63 (10.05) | 15 (8.77) | 11 (8.80) | 31 (11.83 | 6 (8.70) |
| **Trust of information found online** | | | | | |
| Not at all | 12 (1.91) | 2 (1.17) | 3 (2.40) | 5 (1.91) | 2 (2.90) |
| Slightly | 177 (28.23) | 60 (35.09) | 30 (24.00) | 63 (24.05) | 24 (34.78) |
| Moderately | 390 (62.20) | 96 (56.14) | 83 (66.40) | 171 (65.27) | 40 (57.97) |
| Very much so | 48 (7.66) | 13 (7.60) | 9 (7.20) | 23 (8.78) | 3 (4.35) |
| **Most trusted sites (select up to 3)*** | | | | | |
| Social media | 56 (8.93) | 14 (8.19) | 13 (10.40) | 19 (7.25) | 10 (14.49) |
| Official NHS website | 490 (78.15) | 128 (74.85) | 97 (77.60) | 212 (80.92) | 53 (76.81) |
| Charities | 401 (63.96) | 114 (66.67) | 87 (69.60) | 162 (61.83) | 38 (55.07) |
| Scientific literature | 175 (27.91) | 55 (32.16) | 29 (23.20) | 64 (24.32) | 27 (39.13) |
| Websites of medical bodies | 325 (51.83) | 89 (52.05) | 64 (51.20) | 133 (50.76) | 39 (56.52) |
| Online news sites with reports about the menopause | 47 (7.50) | 13 (7.60) | 6 (4.80) | 26 (9.92) | 2 (2.90) |
| Other | 28 (4.47) | 7 (4.09) | 4 (3.20) | 16 (6.11) | 1 (1.45) |

Key. * Total percentages may equal more than 100% as participants were able to select more than one answer option; NHS: National Health Service

**Table S13.** Themes and codes identified from Thematic analysis. Total percentages may exceed 100% because multiple codes/themes can apply to a single data point. Percentages were calculated by dividing the frequency of each code by the total number of responses (n=394).

| **Theme** | **Codename** | **Code description** | **Illustrative quote(s)** | **Frequency** | **%** |
| --- | --- | --- | --- | --- | --- |
| **Information quality and accessibility** | Request for research | Perceived lack of research into the menopause (e.g., secondary health outcomes, treatment or management options and their efficacy), as well as a request for more accessible research information and data | *"I'd like to be more familiar with research into the menopause ."  "There is some data and scientific literature on and not-hormonal interventions. They are a hard read and not easy to translate to practical actions to take. Most of the products are lacking scientific evidence. There is a large scope to provide credible and applicable advice on non-HRT treatments and products."  "Detailed research evidence that shows If taking testosterone can improve symptoms of the menopause and how this can be accessed in the UK."* | 30 | 7.61 |
|  | Information perceived as generic or vague | Available information related to the menopause is perceived as generic or vague | *"Most info is aimed at the majority"  "More specific information,instead of maybes or might be."* | 14 | 3.55 |
|  | Distrust of information | There is a distrust of information available related to the menopause | *"It is difficult to figure out what to trust/believe"* | 14 | 3.55 |
|  | Information perceived as outdated | Available information related to the menopause is perceived to be outdated | *"Official resources (e.g. NHS websites) can be outdated"* | 11 | 2.79 |
|  | Information is difficult to understand, or website is difficult to use | Available information related to the menopause is considered difficult to understand or available websites are difficult to use or navigate | *"I would like it all explained in a simpler way, as having brain fog made it hard for me to take it in."  "Broken down into easy reading would help trailing through the minefield of information is draining"* | 9 | 2.28 |
|  | Information is overwhelming | Available information related to the menopause is considered overwhelming | *" it's very overwhelming to know what to trust"* | 9 | 2.28 |
|  | Request for a centralised menopause information hub | A request for a single source which can be relied on to find information related to the menopause | *"One site that has all the information, instead of being sent from site to site"  "A site specifically about menopause with symptoms, what type of treatments, and finding help under one site."  "Something akin to the diabetes UK website."* | 8 | 2.28 |
|  | Request for information source transparency | Importance of having the source of information (e.g., research by an academic institution, research by a company) provided | *"It would be really helpful to have a 'approved by the BMA' standard or something like that so you know what you're seeing is credible."  "I would like to see more accreditation and for people to not be able to make claims without sound evidence. Too many people trying to sell to us a quick fix"* | 7 | 1.78 |
|  | Information is perceived as a sales pitch | Perception that information about treatments/supplements is not independent, and is a sales pitch | *"[…] other resources have an agenda at best (or at worse 'commercialise' menopause, the new buzzword to market products and make money)."  "Feel like I get sucked into social media ads targeted at 50+ year old women trying to sell me dubious supplements."* | 7 | 1.78 |
|  | Information perceived as contradictory | Available information related to the menopause from different sources is contradictory | *"Clarification. Different sources have different information"* | 4 | 1.02 |
|  | Information perceived as patronising | Perception that available information related to the menopause is patronising | *"For the information to be less patronising"* | 3 | 0.51 |
|  | Request for website filters for personalised information | Request for a website filter function to quickly identify the most personally relevant information | *"customised suggestions"  "A way to filter the information so it can be made more specific to my individual needs & experiences."* | 2 | 0.76 |
| **Treatment** | Decision support tools for treatment | Request for simple information, guidance and decision support tools (e.g., a flowchart) for care pathways and treatment or management options | *"options available and how to decide what's right for you"* | 53 | 13.49 |
|  | Request for information about alternatives to HRT | Request for information on what to do if you cannot or don't want to take HRT | *"Treatment other than medication for people who can't use HRT"* | 36 | 9.16 |
|  | Request for information about menopause treatment and pre-existing or co-occurring conditions | Request for more information on the cancer risk associated with HRT or for information about taking HRT during/after receiving treatment for cancer or other conditions (e.g., gynecological conditions, blood clots, stroke) | *"Specific info & treatments for someone who has had triple negative breast cancer"* | 26 | 6.62 |
|  | Request for information about testosterone | Request for information about testosterone as a menopause treatment | *"More info about testosterone. It seems to be vitally important but there is very little info/ research available."  "Detailed research evidence that shows If taking testosterone can improve symptoms of the menopause and how this can be accessed in the UK."* | 16 | 4.07 |
|  | Request for menopause treatment safety information | Request for more information on the safety of menopause treatments | *"Warning of side effects"  "Comprehensive treatment both medical and complementary as well as side effects"* | 17 | 4.33 |
|  | HRT misinformation | Perceived misinformation about HRT | *"Online resources should make it clear […] that HRT is not so incredibly dangerous."  "Accurate information about the health BENEFITS of [HRT] and removal of the hugely out of date and debunked 'scare stories' about HRT."* | 8 | 2.04 |
|  | Request for information about hormonal contraception | Request for information on hormonal contraception during the perimenopause/menopause | *"More on effect of combined pill and coming off this during peri menopause"* | 5 | 1.27 |
|  | Request for diversification of treatment guidelines | Request for treatment guides (e.g., NICE guidelines) to address perceived lack of guidance of management of medical/surgical menopause or "atypical" menopausal presentations | *"There is no NICE guidance on risks of HRT post surgery for endometrial cancer and very little research."* | 4 | 1.02 |
|  | Request for information related to the consequences of no treatment | Request for information about consequences of not taking any menopause treatment (i.e., letting the menopause happen "naturally") | *"What if you don't do anything at all and let it all happen naturally, what are the consequences of this"* | 2 | 0.51 |
| **Groups lacking information** | Request for information available for HCP(s) | Request for improved training for HCP(s) in terms of education or attitude/approach, or access to informational resources tailored to HCP(s) | *"Resources to take to your GP if they are not up to date on the menopause."  "Better training of GP's to understand and support us."* | 14 | 3.56 |
|  | Requestion for menopause information/guidance for workplaces | Request for menopause information available for and tailored to workplaces/managers | *"More online resources for employers to access."* | 12 | 3.05 |
|  | Request for information specific to medical/surgical menopause | Request for information specific to medical and surgical menopause | *"More recognition about surgical menopause and the impact of symptoms"* | 12 | 3.05 |
|  | Request for menopause information/guidance for partners | Request for menopause information available for and tailored to partners or specific to relationships | *"Menopause should be taught to both women and men. I had to make my husband read others experiences so that he understand my plight and the help I needed."  "Relationship help"* | 8 | 2.04 |
|  | Request for menopause information/guidance for men/boys | Request for menopause information available for and tailored to boys and men | *"Educate boys and men, it's important"* | 7 | 1.78 |
|  | Information perceived as tailored to specific population | Perception that online information is tailored towards certain groups (e.g., "healthy" women) | *"Much seemed geared towards "normal" healthy women - ie, not fat like me, or with pre-existing depression."* | 4 | 1.02 |
|  | Request for menopause information/guidance for young people | Request for menopause information available for and tailored to young people | *"Education from puberty for girls about the impact and expectations."* | 3 | 0.76 |
|  | Request for information about neurodivergence | Request for more information about menopause in neurodivergent women | *“Menopause for neuro divergent women.”* | 1 | 0.25 |
| **Symptom specifics** | Request for information about the full list of possible symptoms | Request for a full list of all possible perimenopause and menopause symptoms, as well as more information available about symptoms of the perimenopause and menopause (e.g., sexual symptoms, genitourinary symptoms) | *"More information about ALL the symptoms and effects that the menopause has on a woman's body. Not every woman is affected by all or even some of the symptoms."* | 57 | 14.50 |
|  | Request for information to help identify the perimenopause/menopause | Request for information on how to recognise or "spot" that you may now be in the perimenopause/menopause | *"How to tell you are peri menopausal"* | 16 | 4.07 |
|  | Request for information/advice for mental health/psychological symptoms | Request for more information/advice for mental health/psychological symptoms related to the menopause | *"More information about the psychological symptoms and how to manage them. Most information is heavily swayed towards the physical symptoms. Not enough help or information about the psychological side. "  "Clarity to women in their 40's about the beginnings of menopause as I was completely unaware that this is what I was going through."* | 15 | 3.82 |
|  | Request for information about chronicity of symptoms | Request for information about which perimenopause/menopause symptoms are permenant and which are temporary, and whether symptoms alleviate in the post-menopausal stage | *"There's no information about what symptoms are permanent and what are transient. For example, is this my lot for the rest of my life? Will I ever get my sex drive, energy and motivation back? Will I get my confidence back? Will I ever get my previously fast brain back or will I always feel inadequate. Before menopause I thought symptoms were transient but now it seems they are permanent and it makes me think 'if this is me now, what's the point of living'."* | 15 | 3.82 |
|  | Request for information about hormones | Information related to the recognition, understanding, or explanation of the role of hormones in health and the menopause | *"What happens after menopause, how hormones balance ."  "More about the effects of low testosterone in women"* | 8 | 2.04 |
|  | Request for information about menopause symptom prevalence | Request for more information on symptom prevalence | *"Perhaps more data rather than the 'you might experience' just to get some idea about how prevalent particular symptoms are."* | 3 | 0.76 |
|  | Request for information to differentiate memory problems and dementia | Request for information on how to differentiate between menopausal related memory problems and dementia | *"Memory issues v dementia"* | 1 | 0.25 |
| **Health care access and support (including offline)** | Request for information about when to seek professional care | Request for advice on when to seek professional care and the importance of online resources providing signposting to professional care when appropriate | *"I would love to know the tipping point at which you should go and talk to your gp"  "When is [it] time to seek medical advice without feeling you are wasting their time."* | 23 | 5.85 |
|  | Importance of access to menopause specialists | Importance of having access to menopause specialists, online and in-person | *"How to refer oneself to specialist services"* | 19 | 4.83 |
|  | Request for advice on discussing menopause with HCP(s) | Request for information/advice on discussing menopause with HCP(s), including how to advocate for oneself | *"what to do when your GP wont prescribe what you think you need, in my case a higher dose of oestrogen"  "How to approach a GP who seems poorly educated on menopause."* | 18 | 4.33 |
|  | Importance of access to HCP(s) | Importance of being able to have access to HCP(s) | *"Not online.* *However there should be medical professionals to provide more information, reassurance."* | 4 | 1.02 |
|  | Request for information about how to gain a referral | Request for information about how to gain a referral to a specialist HCP | *"How to refer oneself to specialist services"* | 3 | 0.76 |
|  | Dislike of Private Providers | Dislike of private providers for menopause care | *"I'd like to see NHS services instead of private providers."* | 3 | 0.76 |
|  | Request for online chat with HCP(s) | Request for a in-website "chat" function to connect with health care professionals | *"Live chat with a trained and qualified practitioner"* | 2 | 0.51 |
|  | Perceived "postcode lottery" of care | Care provision/availability is perceived as a postcode lottery | *"Explanations why some women cannot have HRT and more details around this. I have no real understanding of this treatment but have. Even told I cannot have it! Why? Is it true about location lottery ?"* | 2 | 0.51 |
|  | Blood test misinformation | Perceived misinformation about blood test to test for menopause | *"More information on perimenopausal symptoms and that FSH blood tests aren't absolute. "* | 1 | 0.25 |
| **Other support outside of HCP** | Request for information on getting support in the workplace | Request for information on how to get menopause support in the workplace | *"Knowing my rights at work and what help I can get"  "More information about employee rights and employer responsibilities w[ith] regard to support"  "i have a younger boss whose mother sailed through so my symptoms were dismissed referred to OH who were ignored and now i have resigned as i felt i had no choice"* | 14 | 3.56 |
|  | Request for directory of support groups | Request for directory of or help finding support groups | *"Support groups in my area"  "More local support groups"* | 11 | 2.80 |
|  | Perceived utility of online support groups | Perceived usefulness of online support groups | *"what I found most helpful was some facebook groups where women shared their experiences and stories as then there was always something you could relate to and understand how they'd managed or what advice they gave or had found etc."* | 6 | 1.53 |
|  | Request for signposting to apps | Request for signposting to relevant apps for the menopause | *"More signposting for things such as the Balance app"* | 1 | 0.25 |
|  | Request for information on support group benefits | Request for information on the benefits of menopause support groups | *"Yes support group data"* | 3 | 0.76 |
|  | Request for a menopause support phone service | Request for a menopause support line via telephone | *"A menopause support line would be great."* | 2 | 0.51 |
|  | Request for online symptom checker | Request for an online symptom checker with treatment recommendations | *"A symptom checker with treatment recommendations"* | 1 | 0.25 |
| **Lifestyle support and self-management** | Request for information about non-formal menopause management | Request for information, advice or recommendations for non-formal management options of menopause symptoms not accessed via a HCP (e.g., homeopathic options, supplements, lifestyle changes) | *"naturally treat/support hormonal changes with the appropriate supplements"  "More expert by experience information and more focus on lifestyle changes and natural remedies"* | 27 | 6.87 |
|  | Request for information about food, diet, exercise, and weight management for menopause | Request for information about specific food or diet and exercise for menopause, as well as information related to weight loss or management | *"Recommendations on food types that can help symptoms."  "any specific food/diet to help with the symptoms"* | 20 | 5.09 |
|  | Request for information about menopause products and supplements | Request for more information about menopause product and supplements, including reviews | *"Supplements that actually work , so many offer so much , but don't deliver"  "Reviews of natural supplements etc"  "More information about intimate moisturiser."* | 16 | 4.07 |
|  | Request for information about vegan medication | Importance of highlighting information that menopause medication may not be vegan and getting vegan medication | *"Types of HRT that don't have mares' urine as an ingredient. I am not anti animal product, but I do not like the idea of the mares being kept in small stalls as well as being catheterised."* | 2 | 0.51 |
| **Menopause and co-occurring conditions** | Request for information about menopause and pre-existing conditions | Request for more information about the association between menopause and pre-existing conditions or co-occurring conditions (e.g., physical health conditions, mental health conditions, gynecological conditions) | *"More advice about how the menopause can worse existing general anxiety disorder"  "It is impossible to find any information for women who suffer with PCOS and how to manage this and the menopause at the same time and they interact with each other. We are in desperate need of guidance and support for PCOS & Menopause."  "Pmdd and it getting worse before menopause ."* | 24 | 6.11 |
|  | Request for support for infertility | Request for information and support in regard to infertility related to the perimenopause and menopause | *"Some help with becoming infertile in your 30s. There was no offer of counselling or egg freezing."* | 1 | 0.25 |
| **Patient empowerment** | Request for narrative accounts | Request for more lived experience accounts | *"I would like to see more women's personal experiences."  "Women discussing interacting and discussing their menopause experiences"  "Personal stories of women coping and how they cope"* | 15 | 3.82 |
|  | Request for reassurance | Request for online information to provide reassurance about the menopause experience | *"How normal it is to talk about it, that you're not alone and that it will get better"* | 7 | 1.78 |
|  | Role of celebrities | Importance of celebrities talking publicly about the menopause, and the perception of the celebrities or public figures in communicating information related to the menopause (i.e., trust vs. distrust) | *"Advice NOT from celebrities who seem to be making money from it."* | 4 | 1.02 |
|  | Information is perceived as negative | Perception that information available is overly negative/scaremongering | *"I came across what I can only describe as negativity and horror stories."   "All quite negative and scary, which had not mirrored my actual experiences."* | 4 | 1.02 |
|  | Request for information highlighting positive menopause experiences | Request for positive aspects of the menopause and narratives of people who have had positive menopausal experiences | *"More positivity"  "Happiness. Its not all doom and gloom."* | 4 | 1.02 |
|  | Use of negative images | Perception that online resources for information related to the menopause use images of older and unattractive women | *"I'd like for menopause information givers, to stop using horrible stock images of frumpy women, silver hair and all things ugly and old lady ISH, because it depresses me and makes me feel ashamed and horrified about getting older and being menopausal."* | 1 | 0.25 |
|  | Request for financial advice | Request for financial support or advice related to the menopause | *" maybe some financial support for alternative therapies in these situations. I'm currently spending a lot of money to try to get through."* | 1 | 0.25 |
| **Perimenopause** | Perceived lack of perimenopause information | Perceived lack of or request for information about the perimenopause | *"I struggled to find much info[rmation] on the perimenopause"* | 18 | 4.58 |
|  | Request for information to differentiate perimenopause and menopause | Request for more information on how to differentiate between perimenopause and menopause | *"More help to identify issues to differentiate between perimenopause and menopause"* | 4 | 1.02 |

Key. HCP(s): Health care professional(s)
